# Evolution of population structure in a commercial European hybrid dent maize breeding program and consequences on genetic diversity

**DOI:** 10.1007/s00122-025-05008-5

**Published:** 2025-08-30

**Authors:** Romain Kadoumi, Nicolas Heslot, Fabienne Henriot, Alain Murigneux, Mathilde Berton, Laurence Moreau, Alain Charcosset

**Affiliations:** 1 Limagrain Field Seeds, 28 Route d’Ennezat, 63720 Chappes, France; 2 Université Paris-Saclay, INRAE, CNRS, AgroParisTech, Génétique Quantitative et Evolution (GQE) - Le Moulon, 91190 Gif-Sur-Yvette, France

## Abstract

**Key message:**

Differentiation between Stiff Stalk and Non-Stiff Stalk heterotic groups increased significantly over time, while genetic diversity within both groups declined, highlighting the impact of long-term selection in hybrid maize breeding.

**Abstract:**

Differentiation between Stiff Stalk and Non-Stiff Stalk heterotic groups increased significantly over time, while genetic diversity within both groups declined, highlighting the impact of long-term selection in hybrid maize breeding. The separation of germplasm into complementary heterotic genetic pools is fundamental to modern hybrid breeding programs. This approach facilitates the development of high-performing hybrids by maximizing heterosis through crosses of divergent inbred lines. Maintaining heterotic structure ensures continuous genetic gain and selection of divergent alleles, but introducing novel germplasm is equally important to mitigate the risks of diversity loss from repeated selection of elite material. This study presents a large-scale assessment of the evolution of genetic diversity, population structure, and differentiation between heterotic groups, within a private European hybrid dent maize breeding program. Forty years of breeding data and 84,000 genotypes were used. Clustering methods revealed two main heterotic groups in modern germplasm: Stiff Stalks and Non-Stiff Stalks. These two groups originated from Stiff Stalk, Iodent, and Lancaster founders, forming three ancestral groups. Differentiation between heterotic groups was low for early founder inbreds and increased over time. Consistently, intragroup diversity decreased over time, and marker fixation and linkage disequilibrium increased. The main cause of diversity loss germplasm-wide was the merging and genetic homogenization of the ancestral Iodent and Lancaster groups into the modern Non-Stiff Stalk heterotic group. Insights into the genetic relationship between hybrid heterotic group population structure and intragroup diversity can assist breeders in enhancing heterotic group divergence, while preserving diversity across selection cycles. This study provides an overview of the evolution of key genetic metrics, to inform strategies for managing diversity and differentiation in commercial hybrid breeding programs.

**Supplementary Information:**

The online version contains supplementary material available at 10.1007/s00122-025-05008-5.

## Introduction

Maize *(Zea mays subsp. mays)* is a cornerstone of global agriculture, ranking first among cultivated cereals in terms of production and second in acreage worldwide (FAO 2022). Following its domestication in the Americas and subsequent dissemination across the globe, maize was primarily cultivated as open-pollinated varieties (OPVs), also referred to as landraces (Tenaillon and Charcosset 2011; Mir et al. 2013). These OPVs were selected for local adaptation to abiotic and biotic stresses, as well as for traditional uses and regional agricultural practices (e.g., Pyrenees-Galicia flint maize landraces like Aleu, Bareilles, or Lacaune) (Troyer 1999; Beigbeder and Carraretto 2018).

The invention of hybrids and early observation of heterosis (i.e., hybrid vigor) by East and Shull in the early 1900s (East 1907; Shull 1908) marked a turning point in maize agricultural practices and breeding. Maize inbred lines, created from controlled self-pollination of American Corn Belt Dent landraces (CBD) (i.e., a blend of dent and flint types), were developed and led to the production of the first pure-line hybrids (Anderson 1944; Anderson and Brown 1952; Wallace 1956). Compared to the previously established traditional OPVs, selected hybrids exhibited significantly superior performances, greater uniformity, and reduced susceptibility to environmental variability (Crow 1998; Troyer 2006). To systematically exploit the heterosis effect and prevent inbreeding depression, parental inbreds of hybrids need to be unrelated and divergent. This requirement of dissimilarity laid the theoretical foundation for the concept of heterotic population structure (Melchinger et al. 1991; Reif et al. 2005). The organization of North American germplasm in consistent heterotic groups, for efficient selection of inbreds, was proposed at the 1949 annual North Central Regional Corn Improvement Conference and applied in the following years (Tracy and Chandler 2006). The first attempts at inbred line separation were realized according to the geographical and phylogenetic history of CBD landraces. This process relied heavily on the crossing pattern Reid Yellow Dent (Southern Dent types)–Lancaster (Other dents and Northern flint types) (Anderson 1944; Smith et al. 2004; Tracy and Chandler 2006).

The development and wide success of the Iowa Stiff Stalk Synthetic (BSSS), by Sprague (1946), a 16-line synthetic population composed mainly of Reid Yellow Dent heritage, highlighted the interest in tester-based reciprocal recurrent selection (RSS), which had been conceptualized by Hull (1945). It was rapidly adopted by private and commercial programs and contributed to their growing dynamics (Hallauer et al. 1983; Gracen 1986). This breeding method, also named modified reciprocal recurrent selection (MRRS), involves the cyclical and simultaneous selection of two genetically divergent elite groups. Each group of inbreds serves as source material for within-group selection (i.e., intragroup improvement) and provides testers for hybrid selection in the other divergent population (i.e., intergroup improvement). The outcome of this scheme is the creation of a distinct heterotic structure between groups, with improved performances (e.g., yield, adaptive traits, etc.) through the stacking of favorable divergent alleles, and increased genetic differentiation (Comstock et al. 1949; Menz Rademacher et al. 1999; Hinze et al. 2005; Edwards 2011).

Applied to Iowa State University breeding schemes, MRRS yielded pivotal elite public inbreds, now recognized as key dent founders, such as B14, B37, and B73 (Gracen 1986; Troyer 1999; Mikel 2011). The superior performance and seed-producing quality of these elite BSSS inbreds prompted their designation as the primary female group, forming the basis of the Stiff Stalk (SS) heterotic group (Walters et al. 1991). Inbreds combining effectively with Stiff Stalk to yield high-performing hybrids (e.g., B73 x Mo17) were classified as male and placed accordingly in the Non-Stiff Stalk (NSS) group (i.e., formalizing SS–NSS heterotic pattern) (Messmer et al. 1991; Smith et al. 2004; Tracy and Chandler 2006). Consequently, the Non-Stiff Stalk group incorporates diverse inbreds from varying lineages and backgrounds, including Lancaster Sure Crop (LAN) elite lines (e.g., C103, Oh43, Mo17), the Iodent (IDT) group, and other populations, or synthetics (Troyer 1999; Mikel 2008, 2011).

In modern American temperate maize breeding, various strategies for managing heterotic structure have been proposed and implemented in commercial hybrid breeding programs. Most of these approaches are variants of the Stiff Stalk and Non-Stiff Stalk heterotic pattern (Duvick et al. 2003; Reif et al. 2005). Despite their highly structured and distinct nature, modern heterotic groups originate from a small number of close historically dominant populations and public elite inbreds, referred as founders (Smith et al. 2004; Reif et al. 2005). Heterosis and heterotic groups have undoubtedly contributed to increased performances and sustained genetic gain (Penny and Eberhart 1971; Crow 1998). However, the continued emphasis on increasing divergence and the use of tester-based RRS on elite material have led to reduced genetic diversity within the active breeding germplasm (Technow et al. 2021; Smith et al. 2022). This narrowing of diversity could limit genetic gain and the ability to respond to biotic and abiotic stresses in the mid- to long-term (McCouch et al. 2013; Mickelbart et al. 2015; Sanchez et al. 2024).

Implementation of hybrid maize breeding in Europe after WW2 reveals interesting features complementary to those of North America. At European southern or mid-latitudes, modern elite European genetics are largely similar to the ones used in North American temperate genetics, especially dent European ones. This similarity is notably linked to substantial recent transfers of material between private companies and the use of American exPVP lines (Smith et al. 2022). Conversely, at Northern latitudes, colder climates call for earlier flowering and adaptation to cold and humid springs (Unterseer et al. 2016). This constraint has led to the use of European flint genetic landraces as source materials for new inbred lines, which proved excellent partners of North American dent lines, leading to the flint-dent heterotic pattern. This heterotic pattern is currently used mostly for silage purposes in Northern Europe. European flint landraces relate to introductions from Caribbean and Northern flint landraces into Europe. From their transfer on the European continent, the material underwent severe bottlenecks inherent to the production constraints of early flowering and cold tolerance (Tenaillon and Charcosset 2011). This led to flint European material being sufficiently divergent from American founder gene pools to appear as separate genetic groups (Rincent et al. 2014). So, whereas hybrid breeding mostly shaped heterotic groups in Northern America, North European heterotic groups capitalize on a clear pre-existing population structure (Camus-Kulandaivelu et al. 2006).

Although the development of commercial breeding practices and the evolution of genetic diversity and differentiation are well documented for North American germplasm (Duvick et al. 2003; Smith et al. 2004; Feng et al. 2006; Mikel and Dudley 2006; Nelson et al. 2008; Van Inghelandt et al. 2010; Mikel 2011; van Heerwaarden et al. 2012; Romay et al. 2013; Schaefer and Bernardo 2013b; Beckett et al. 2017; White et al. 2020), it remains underexplored in European material (Allier et al. 2019), notably in European dent-focused breeding programs.

In this study, we present a large-scale analysis of the evolution of population structure of a private European hybrid dent maize breeding program and its impact on heterotic group divergence and the intragroup genetic diversity. We aim to: (1) characterize the evolution of population structure and heterotic group management within a breeding program, tracing changes from founder lines to contemporary experimental selection lines; (2) assess genome-wide and locus-specific genetic diversity alongside group differentiation metrics; and (3) evaluate temporal intra- and between-group dynamics, using a large dataset from a private hybrid breeding program. Insights from this study will provide a comprehensive overview of a European-centered hybrid dent breeding program and offer actionable metrics to manage genetic diversity effectively in inbred development breeding schemes, while maintaining and enhancing heterotic group divergence for optimal hybrid performance.

## Material and methods

### Plant material and genotyping

We analyzed the complete dataset from a European hybrid dent maize breeding program managed by Limagrain Field Seeds. This breeding program follows the Stiff Stalk–Non-Stiff Stalk heterotic pattern and focuses on high-yielding grain varieties for mid-early to mid-late maturity classes, with an FAO maturity rating of 330–500 (European maize relative maturity system; equivalent to relative maturity ratings of 90–105). The main geographical targets for this maturity range include southern France, Central, and Eastern Europe, with specific emphasis on Hungary, Romania, Ukraine, and southwestern Russia. The inbred line development breeding scheme is structured around a 5-year-long tester-based MRRS cycle.

The experimental material includes 1,342 inbreds (i.e., lines which finished the selection cycle and have been used for hybrid varieties development and/or as parents for new breeding cycles) and 81,697 additional selection lines (i.e., lines which have been/are still in the inbred line development pipeline and used exclusively for experimental hybrid crosses). Together, these lines were evaluated across multiple locations in Eastern and Western Europe from 2013 to 2023 and contributed to 85,673 tested hybrids, in over 800.000 plots. These were incorporated into past and current Genomic Selection (GS) calibrations (Meuwissen et al. 2001). To facilitate comparisons with older inbreds and founder lines, the pedigree of each inbred was recovered. All available ascendant parents were retrieved, representing 1,802 additional inbreds. These additional inbreds predominantly consist of maize dent founders—including public elite lines from BSSS (e.g., B14, B37, B73, B84), Lancaster Sure Crop (e.g., C103, Oh40B, Mo17), and other lineages, alongside ex-PVP lines from American seed companies (Gerdes et al. 1976; Mikel 2011)—and Limagrain derivatives from these inbreds. Thus, in the following article, the term “genotypes” is used to characterize the complete dataset of 84.841 genotyped inbred lines (i.e., inbreds, selection lines, and additional inbreds obtained from the pedigree).

Genotyping was performed on the complete set of genotypes using the Limagrain-designed Affymetrix genotyping array, containing 18,480 single nucleotide polymorphisms (SNP) markers (Supplementary Figure S1). The 18 K Affymetrix SNP array is based on the Illumina MaizeSNP50 Genotyping BeadChip, with the BSSS founder B73 taken as the reference sequence. The selected markers were chosen to represent polymorphism in temperate European germplasm, considering both dent and flint major founder lines. Genotypes with a call rate below 90% or a heterozygosity rate exceeding 15% were discarded. Markers with low call rate (< 90%) were also removed. Heterozygotes genotypes found in non-double haploid (DH) lines were kept. If the line was a DH, heterozygotes markers were removed and assigned missing. The dataset had an average genotyping rate of 96.02% and an average heterozygosity rate of 0.74%. Missing data imputation and genotypic dataset phasing was performed using the software BEAGLE 5.4 (Browning et al. 2018), with default settings and no reference panel. No pruning for minor allele frequency was applied, resulting in a total dataset comprising 84,841 genotypes analyzed across 18,439 markers.

To characterize the evolution of this breeding program, genotypes’ registration year in the internal database was agglomerated in non-overlapping 10 years’ time intervals, called “era,” and distributed as follows (Table 1). Additional inbreds obtained from ascending pedigree information were present mainly between era 0 and era 2. They were agglomerated with proprietary modern inbreds as “inbreds” in the table.

**Table 1:** Characterization of the genotype collection size according to its era and its line type

	**Year interval**	**Number of**			
		Total genotypes	Evaluated Inbreds	Inbreds from Pedigree	Selection lines
ERA 0	Pre-1989	315	0	315	0
ERA 1	1990–1999	525	0	525	0
ERA 2	2000–2009	675	0	675	0
ERA 3	2010–2019	35,908	842	287	34,779
ERA 4	2020–2023	47,418	500	0	46,918

^*^inbreds are composed of the 1342 evaluated inbreds and the 1802 additional inbreds obtained from pedigree data

In the following method sections, unless stated otherwise, all analyses were done in R 4.4.1 (R Core Team 2024), and visualizations were plotted using the R package *ggplot2* (version 3.5.1) (Wickham et al. 2024). Comparisons were computed using the pairwise t-test function, implemented in the source R package *stats* (version 4.4.1). If unspecified, significance threshold considered was *p*-value < 0.001.

### Population structure and dimension reductions methods

Global population genetic architecture and inbred relationships were determined using the Bayesian clustering software ADMIXTURE (version 1.3.0) (Alexander et al. 2009). The analysis was conducted using the complete genotypic dataset of 84,841 individuals, with default parameters and 10 cross-validations for unsupervised clustering at K (number of clusters) values ranging from 2 to 10, 20, and 30. As the marker set was considered representative of the analyzed diversity, no marker was removed due to allele frequencies or linkage disequilibrium.

To estimate the optimal number of clusters (K), Bayesian Information Criterion (BIC) values were calculated using the function *find.cluster()* from the package *adegenet* (version 2.1.10) (Jombart et al. 2023). Population clustering was selected based on the K value with the lowest BIC across 11 tested K-values and on prior global knowledge of dent maize heterotic structure. A low BIC value indicates the best balance between the model’s goodness of fit and its complexity. In consecutive clustering analyses, having the minimum BIC will result in a maximized between-group and minimized within-group variance while conserving a parsimonious model complexity. Cluster assignation and admixture-related metrics were evaluated using two indicators. Cluster Maximum Assignation Probability (CMAP) was calculated for each genotype, ranging from (K)^−1^ (i.e., equal probability assignation across all K clusters) to 1 (i.e., complete assignation to a single cluster). Genotypes were assigned to the cluster with the highest assignation probability, regardless of its value. However, genotypes with a CMAP below than 75% were also classified as “admixed.” Principal component analysis (PCA) was used to identify the largest source of variation in the dataset and to visualize the genotypes genetic landscape. PCA was run with the R package *smartsnp* (version 1.1.0) (Herrando-Perez et al. 2021) and the function *smart_pca()*, which utilizes the *eigenstrat* method (Price et al. 2006). The top five principal component (PC) axes were retrieved.

### Population differentiation, fixation index, and genetic distances

Differentiation between heterotic groups was estimated, on a two populations per-marker basis, using the Weir and Cockerham fixation index (F_ST_) general expressions (formulas 1–4) (Weir and Cockerham 1984), as implemented in PLINK 1.9 (Chang et al. 2015). The global pairwise F_ST_ value was calculated as the genome-wide average. Higher F_ST_ values indicate greater differentiation between two populations. Markers with F_ST_ superior to 0.75 were considered high-F_ST_ markers, and their proportions (noted as P[F_ST_ > 0.75]) were recorded for comparisons between eras.

Genetic relatedness and distances between inbreds were calculated using the Hamming distance (Hamming 1980). This method counts single base pair variations between two sequences, independent of the allelic state (homozygote or heterozygote). It was selected for its straightforwardness, resulting in fast computation and its applicability to single nucleotide polymorphisms analysis (Pinheiro et al. 2012). As the quantity of genotypes renders the calculation of all combinations computationally challenging, i.e., more than 7 billion distances (84,841 × 84,841 distance matrix), marker-based pairwise genetic distances were computed only for inbreds, and not the selection lines.

### Random sampling and variance calculation

To obtain values of deviation from the average metrics, variance was calculated using a random sampling method applied to the Weir & Cockerham fixation index (F_ST_) and mimicking bootstrap process (Efron 1979; Petit and Pons 1998). A subsample was obtained through 10% of the genotypes of the selected case. Sampling was done at random, with replacement. Two hundred replicates were completed for each case. Random sampling protocol was only employed on inbreds to limit sample size bias between eras and reduce the potential relatedness between intragroup genotypes. If selection lines had been kept, multiple cases of full-sibs and half-sibs’ families would have been found in era 3 and era 4, which might have reduced dissimilarity.

### Allele frequency, heterozygosity, and linkage disequilibrium

To assess the levels of genetic diversity inside heterotic groups, multiple genome-wide and marker-specific indicators were used. The change in allele frequency between eras was monitored to assess potential allelic deviation between era 0 and era 4. Genome-wide distribution of the Minor Allele Frequency (MAF) was analyzed. Following the methods of Technow et al. (2021), available allelic diversity of each heterotic group was assessed through the proportion of markers with a MAF below 5% (noted P[MAF < 0.05]). This metric (labeled “Extreme-Tail Marker”) captures SNPs that are close to fixation or extinction and thus have close to null diversity. To complete this metric, additional MAF check indicators were considered: P[MAF > 0.45] for hyper-polymorphic markers and P[MAF < 0.01] for monomorphic markers.

Genetic diversity for each subset was estimated according to Nei and Tajima (1981), using the biallelic system of the gene diversity *H* (also named Expected Heterozygosity H_e_), where $${H}_{e}=2*p*(1-p)$$, and p is the frequency of the major allele. Linkage Disequilibrium (LD) was calculated as a pairwise correlation r^2^, according to Hill and Robertson (1966), using PLINK 1.9 (Chang et al. 2015), with the function *“–r2 –ld-window-r2 0*”. LD was determined for each chromosome, considering all pairwise intrachromosomic SNP marker combinations with a non-null pairwise correlation. For each combination, markers physical distance (bp) was assessed and grouped into a 5-kb non-overlapping window, with a maximum distance of 1 Mb. Linkage Disequilibrium Decay (LDD) graphical representation was realized with the R package *ggplot2* and the function *geom_smooth()*. The smoothing function employed to obtain a LDD trend was a monotone decreasing and convex P-spline (coded “mdcx”) from the R package *scam* (version 1.2–16) (Pya and Wood 2015). In addition, to better characterize LD evolution in time, LD at 0-Mb, LD at 1-Mb, and average LD were determined. Average LD was calculated using the obtained convex mdcx P-spline function between the 0 and 1 Mb distance interval, which provides the LDD Area Under Curve ($${LDD}_{AUC}$$), and average LD was estimated as: $$\overline{{\mu }_{LD}}=\frac{{LDD}_{AUC}}{\text{Distance Covered}}$$ with the distance covered being here 1-Mb (i.e., 1e + 6).

## Results

### Population structure of European dent maize program

**Fig. 1:**
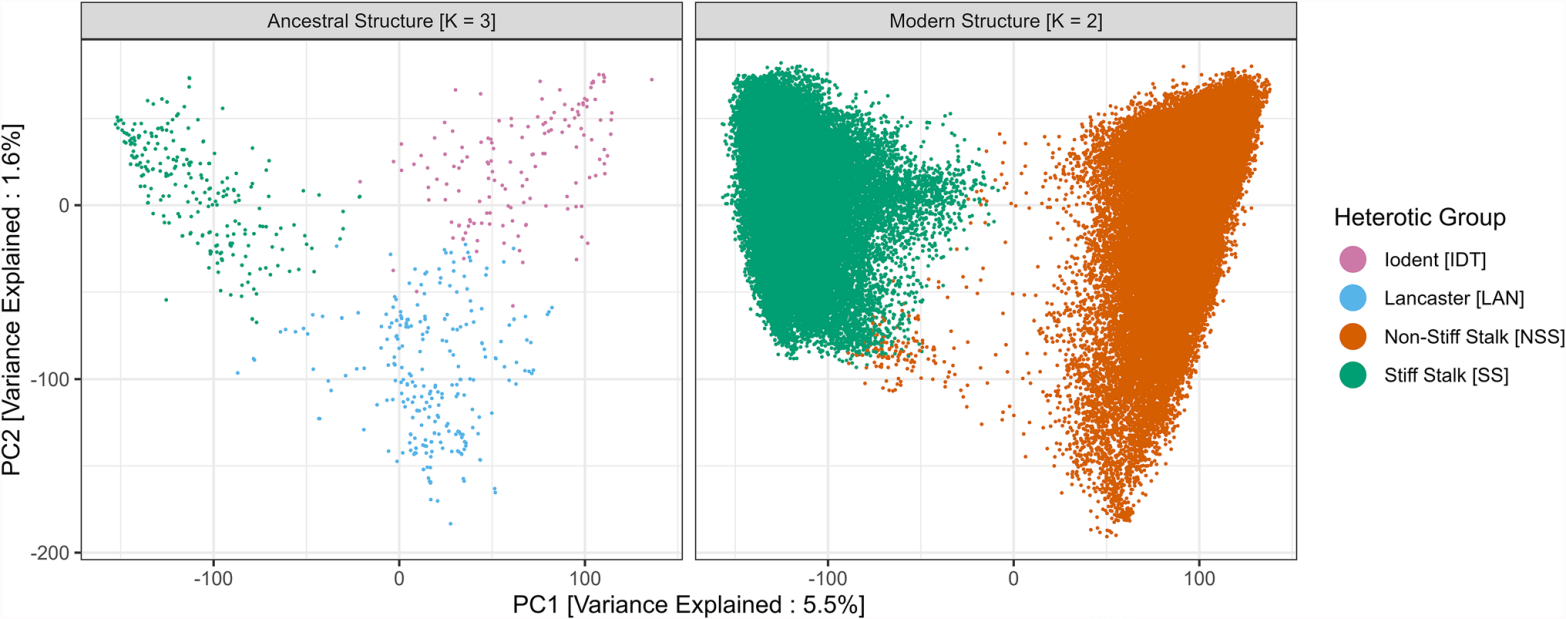
Evolution of the germplasm’s population structure visualized by PCA scatterplots (PC1 and PC2). Both scatterplots were realized on the same source data (hence the similar variance); only a filtering by era was realized. Each dot represents a different genotype. (left) PCA scatterplot filtered for era 0 and era 1 [ancestral structure] (right) PCA scatterplot filtered for era 4 [modern structure]. Color scheme was realized according to the main heterotic group assignation proportion

Principal components analysis highlighted a clear genetic differentiation among genotypes. Observed genetic structure reflects current heterotic structure management, with two main heterotic groups (Stiff Stalks and Non-Stiff Stalks) (Fig. 1). The first two principal components explained 7.1% of the variance across the 18,849 SNP markers, with PC1 (5.5%) separating the global population into SS and NSS, and PC2 (1.6%) separating in a gradient manner the NSS into LAN and IDT subgroups. ADMIXTURE results for low-K parameters support these findings, with K = 2 explaining PC1 and K = 3 explaining PC1 and PC2. These results also indicate the presence of admixed genotypes, intermediate between the two heterotic groups. ADMIXTURE barplot results for K = 3 can be found in the supplementary material (Supplementary Figures S9 to S13).

Germplasm separation into increasing numbers of clusters with the software ADMIXTURE did not lead to a BIC minimum, so that no ideal number of K clusters could be found. We suppose that this relates to the strong pedigree structure of the germplasm. Consequently, to match the current breeding scheme of the SS–NSS heterotic pattern, computation and analysis of population structure metrics were realized using the unsupervised ADMIXTURE results for *K* = 2. Clustering of the 84,841 genotypes for *K* = 2 was balanced, with 56% of them assigned to the NSS side. Average CMAP, which gauges mean genotypes purity to their assigned group, was similar for both heterotic groups, with an average CMAP_SS_ = 0.911 ± 0.087 and an average CMAP_NSS_ = 0.934 ± 0.087. The proportion of genotypes with high admixture (CMAP below 75%) was 5.2%, and the two groups contributed equally on average.

Consideration of the eras in the principal components analysis revealed a weak structuration in the founder population (era 0 and era 1), with a segmentation in 3 sub-clusters (i.e., *K* = 3; SS, IDT, and LAN), noted as “ancestral structure” (Fig. 1). Current modern structure (era 4) reveals the merging of the Iodent and Lancaster sub-heterotic groups into the NSS heterotic group and the increasing separation between the two main pools (SS and NSS) (Fig. 1). Analysis of population structure indicators revealed a decrease in admixed genotypes, with 74.7% of genotypes with less than 75% CMAP in era 0, compared to 3.8% in era 4 (Supplementary Figure S2).

### Analysis of diversity metrics and evidence for a time-dependent decrease

**Fig. 2:**
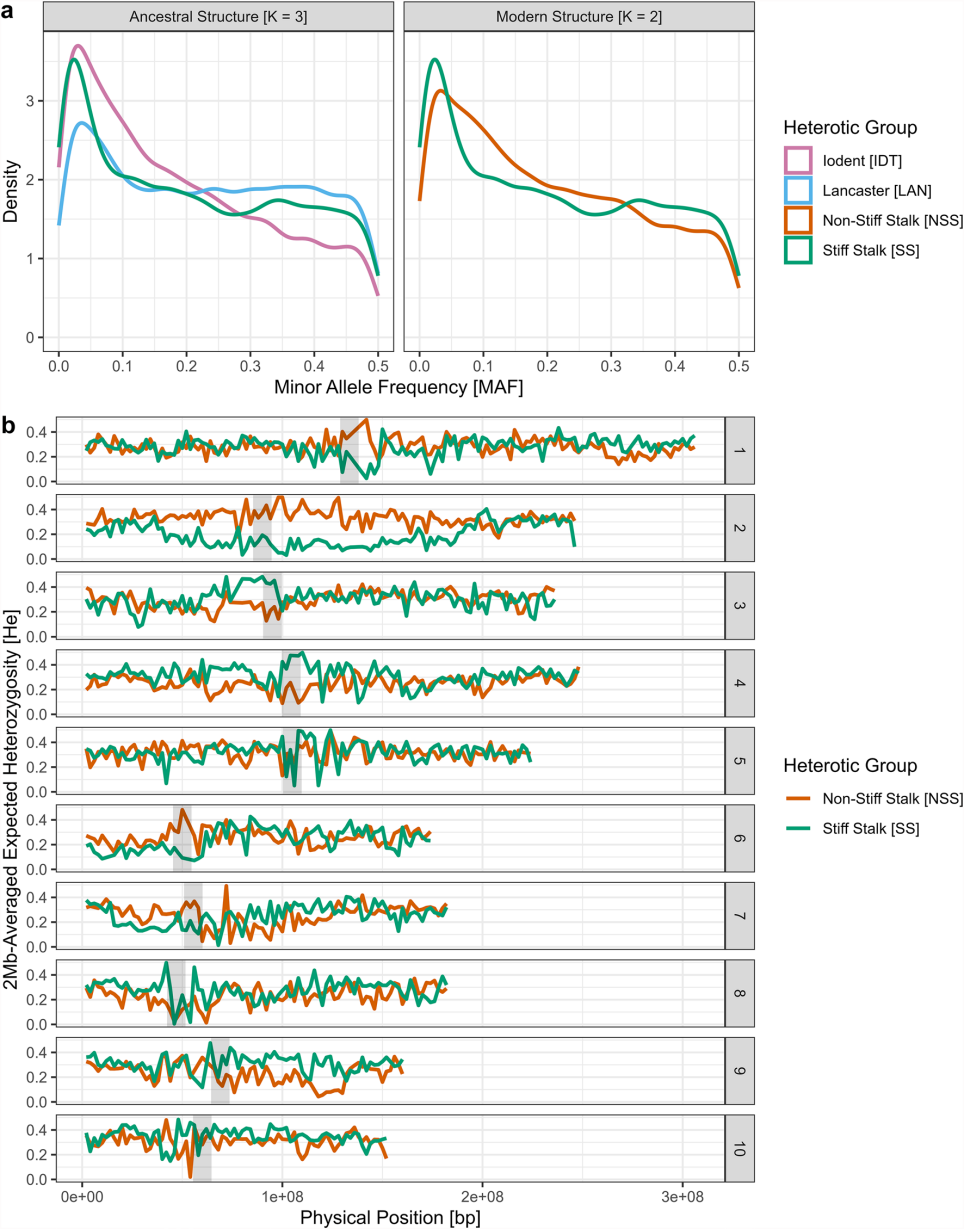
Overview of the intragroup genetic diversity of the hybrid dent maize germplasm **a** Comparison of the MAF density per heterotic group, using different population clustering: ancestral configuration (based on K = 3) on the left and modern configuration (based on K = 2) on the right. **b** Genome-wide representation of the Minor Allele Frequency between the modern heterotic groups. Estimated centromeres position is represented by the gray regions

Analysis of minor allele frequency (MAF) distribution revealed distinct patterns of skewness between heterotic groups. The SS group exhibited an overrepresentation of extreme MAF (both high and low) compared to the NSS group (Fig. 2a). Average MAF and its standard deviation (SD) were slightly higher in the SS (*µ* = 0.213; SD = 0.153) than in the NSS (*µ* = 0.206; SD = 0.144). Characterization of MAF in extreme-tail markers (i.e., P[MAF < 0.05]), using the proposed descriptors of Technow et al. (2021), confirmed this disparity: 25.2% of SNPs in SS had MAF below 0.05, compared to 11.1% in NSS. It is to be noted that among these proportions, 5.5% of these extreme-tail SNPs were shared between both heterotic groups.

The proportion of markers with MAF above 0.45 was also higher in SS (7.8%) compared to NSS (6.5%) (Fig. 2a). Genome-wide analysis of locus-specific expected heterozygosity (He) for the global population showed variability, without a clear positional trend (e.g., centromeric vs. telomeric regions) (Supplementary Figure S3). However, heterotic group-specific He displayed extensive regions of reduced diversity, especially in the SS germplasm, including a 100-Mb-long region on Chromosome 2 (Fig. 2b).

Non-Stiff Stalk segmentation into its ancestral substructure (IDT and LAN), following K = 3 clustering results, shows substantial heterogeneity in allele frequency distributions. The MAF distribution of the IDT substructure was more left-skewed (*µ* = 0.189; SD = 0.142) compared to both SS and LAN (*µ* = 0.233; SD = 0.148) distributions (Fig. 2a). While P[MAF < 0.05] was lower in IDT than that in SS, with 20.1% and 25.2% of markers concerned, respectively, it still represented a significant fraction. In contrast, LAN exhibited a lower rate of extreme-tail markers (P[MAF_LAN_ < 0.05] = 14.83%) and higher MAF across the genome. These values are more in line with obtained NSS results. This suggests that LAN contributed as a significant source of polymorphism and as a key genetic background within the NSS heterotic group. These results differ from the ADMIXTURE origin assignation, where LAN inbreds only represent 19.57% of NSS genotypes.

**Fig. 3:**
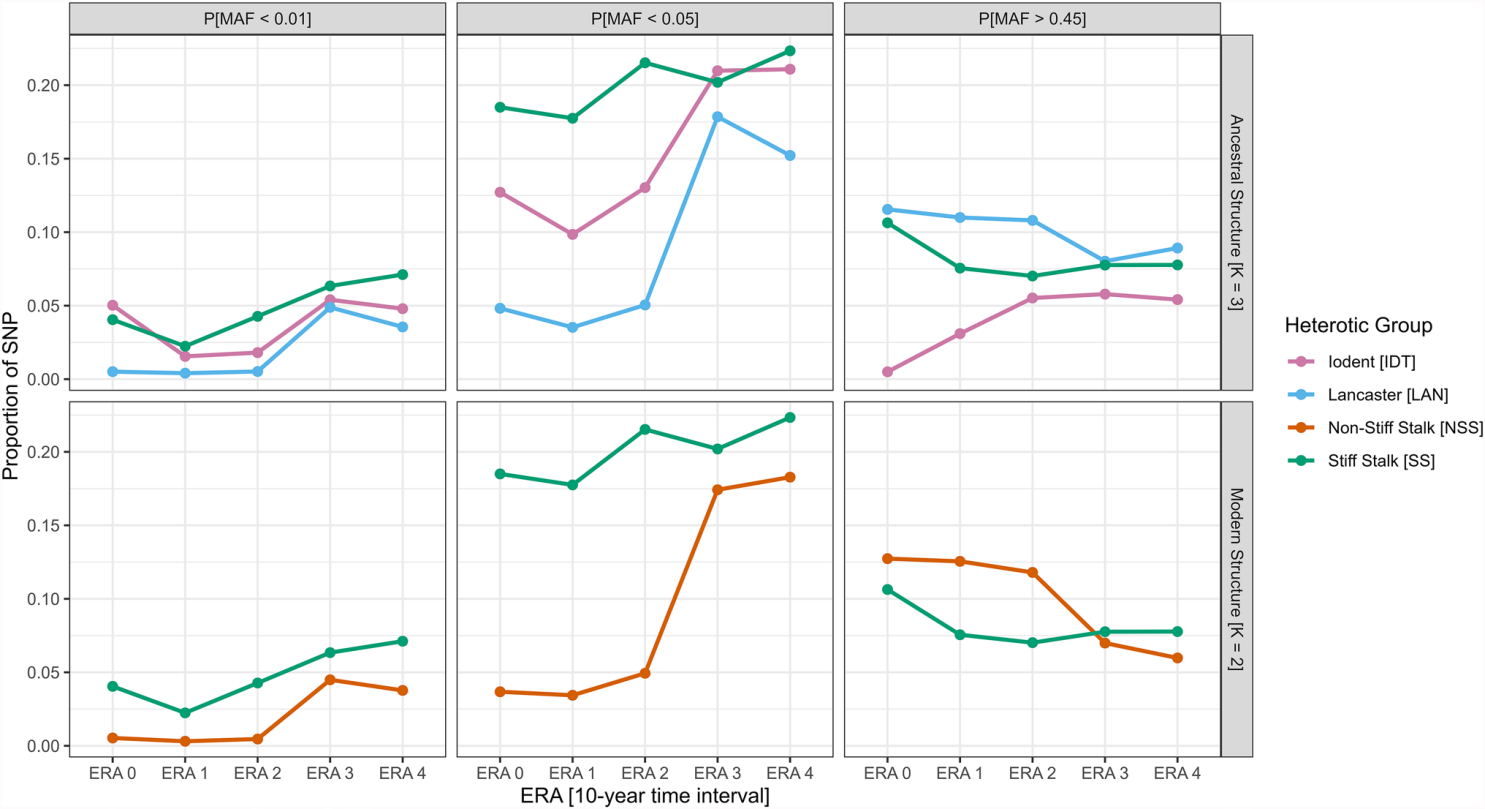
Evolution of the intragroup diversity between era 0 [pre-1990 germplasm] and era 4 [2020 germplasm]. Proportion of SNP markers according to MAF checks at different frequency thresholds, using different population clustering: ancestral configuration (based on K = 3) on top and modern configuration (based on K = 2) on the bottom

Incorporation of the era factor in the analysis revealed a decrease in average He between era 0 (0.383) and era 4 (0.345), independent of the selected heterotic group (Table 2). Individual heterotic groups revealed contrasting trends, with He_(NSS)_ reducing from 0.371 to 0.279 and He_(SS)_ remaining stable (Table 2). Consideration of MAF thresholds at 1%, 5%, and 45% further demonstrated these dynamics (Fig. 3). In accordance with a decrease in average He, NSS proportion of extreme-tail markers increased from 3.68% in era 0 to 18.28% in era 4. Yet, despite stability in SS average allele frequencies across eras, its proportion of extreme-tail markers also increased by 20.76%, reaching 22.3% of SNPs in era 4. This increase in genome fixity can also be noticed through the additional MAF thresholds in both heterotic groups, with an increase in P[MAF < 0.01] and a decrease in P[MAF > 0.45] (Fig. 3). Similar trends can be noticed when using the MAF indicator (Supplementary Table 1).

**Table 2:** Expected Heterozygosity (He) evolution for the global germplasm and for each heterotic group, with the decomposition of the Non-Stiff Stalk into its subclusters

	GLOBAL GERMPLASM	STIFF STALK	NON-STIFF STALK		
			Merged	Iodent	Lancaster
ERA 0	0.383^a^ ± 0.118^b^	0.278 ± 0.169	0.371 ± 0.128	0.235 ± 0.129	0.360 ± 0.134
ERA 1	0.378 ± 0.121	0.284 ± 0.162	0.366 ± 0.129	0.283 ± 0.134	0.359 ± 0.130
ERA 2	0.370 ± 0.128	0.272 ± 0.167	0.352 ± 0.136	0.286 ± 0.146	0.353 ± 0.134
ERA 3	0.348 ± 0.145	0.290 ± 0.168	0.288 ± 0.162	0.267 ± 0.163	0.302 ± 0.166
ERA 4	0.345 ± 0.148	0.281 ± 0.172	0.279 ± 0.159	0.265 ± 0.161	0.312 ± 0.161

^a^mean He; ^b^standard deviation of the He

Evolution of NSS subclusters allelic diversity confirms the observed general dynamic of LAN serving as source of allelic variation, with the subcluster having significant superior He across all eras (Table 2). The difference in average He between the two subclusters was the highest in era 0 (ΔHe_(ERA0)_ (LAN-IDT) = 0.125), where average He_(IDT)_ was the lowest across all eras. It then increased until era 3 included. This convergence in He values between LAN and IDT can be related to two key phenomena: (1) between era 0 and era 1, He_(IDT)_ significantly increased by + 0.048, and (2) between era 2 and era 3, He_(LAN)_ significantly decreased by − 0.051 (Table 2). This is supported by MAF thresholds, notably in era 0, with IDT having the highest proportion of markers with MAF below 0.01 (i.e., P[MAF_ERA0-IDT_ < 0.01] = 0.0503) and LAN having the highest proportion of markers with MAF above 0.45 (i.e., P[MAF_ERA0-LAN_ > 0.45] = 0.116) (Fig. 3). This evolution is supported by the continuous decrease in LAN assignation, which went from 76.8% of NSS inbred in era 0, to 63% in era 1, 47% in era 2, 24% in era 3 and 15% in era 4.

Genome-wide comparisons of locus-specific values (MAF and He, Supplementary Figures S4) along the genome highlighted that the main SS low diversity region on chromosome 2 significantly (p-value < 0.001) decreased in MAF between era 0 (CHR2-MAF_SS_ = 0.216) and era 4 (CHR2-MAF_SS_ = 0.149). Other zones in SS were already close to fixation in era 0. In contrast, MAF of the loci on chromosome 10 appears to have increased (Supplementary Figure S4). Similar analysis applied to the NSS and its subclusters reveals a globally significant (p-value < 0.001) decrease of diversity along the genome between era 0 and era 4, with specific regions disproportionately impacted, notably in a subcluster-specific manner (Supplementary Figures S5).

### Extent and evolution of linkage disequilibrium within maize dent heterotic groups

**Fig. 4:**
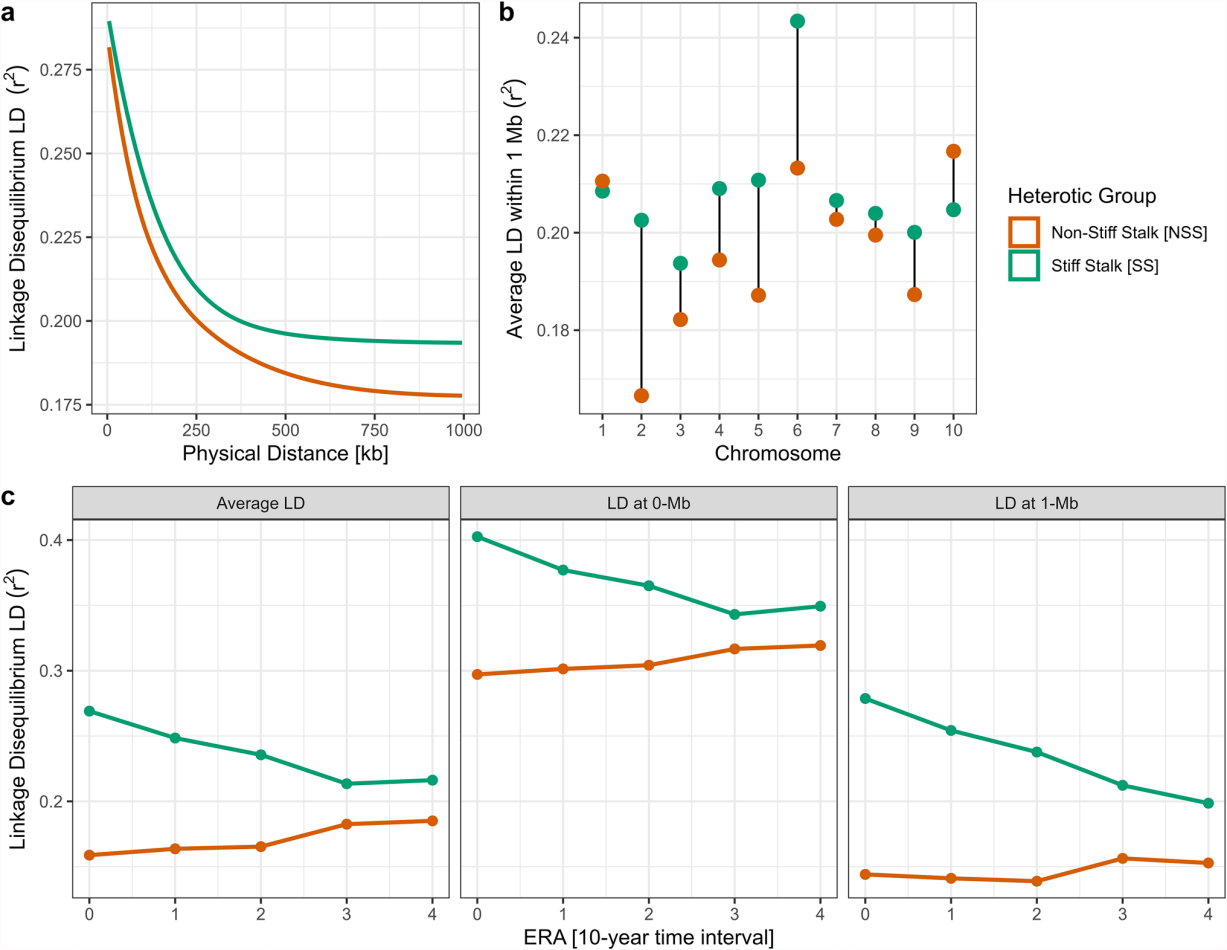
Analysis of Linkage Disequilibrium (LD) and its evolution in the two heterotic groups of the current heterotic pattern. Obtained results are measured by marker pairwise correlation (r^2^). Heterotic groups are shown by distinct colors **a** Line plot of the LD according to the physical distance (in kb) between markers. **b** Dot plot of the average LD within 1 Mb of marker physical distance, for each chromosome. **c** Evolution of LD based on three indicators (average LD within 1-Mb; LD at 0-Mb; LD at 1-Mb)

Linkage Disequilibrium Decay (LDD) results for the SS and NSS heterotic groups, aggregated across all eras, are presented in Fig. 4a. The analysis revealed a significant difference (*p*-value < 0.001) in the rate of LD between the groups. SS genotypes exhibited the highest LD metrics (r^2^_0Mb_ = 0. 337; r^2^_1Mb_ = 0. 194; $\overline{{\mu }_{LD}}$ = 0. 208) compared to NSS (r^2^_0Mb_ = 0.323; r^2^_1Mb_ = 0.178; $\overline{{\mu }_{LD}}$ = 0.195). Chromosome-specific LD comparisons revealed mostly non-significant differences in LD across chromosomes, for both heterotic groups. However, two notable significant exceptions (p-value < 0.001) can be found: higher LD on chromosome 6 for SS and lower LD on chromosome 2 for NSS (Fig. 4b). In accordance with allele frequency trends, inclusion of the era factor demonstrated a significant (*p*-value < 0.001) increase in global LD between era 0 and era 4, independent of the chromosome or the marker pairwise physical distance (Δ(era 4-era 0): Δr^2^_0Mb_ =  + 0.065; Δr^2^_1Mb_ =  + 0.041; Δ $\overline{{\mu }_{LD}}$ =  + 0.078). Further analysis of heterotic group-specific trends revealed that the mean LD ($\overline{{\mu }_{LD}}$) in the NSS rose by 21.8% between era 0 and era 4. In contrast, the evolution of LD in the SS demonstrates decreasing marker pairwise correlations (Fig. 4c).

LD analyses applied to the NSS subclusters and their evolution across eras confirms previous results. Overall, LAN LD (r^2^_0Mb_ = 0.316; r^2^_1Mb_ = 0.176; $\overline{{\mu }_{LD}}$ = 0.188) was significantly lower than both IDT (r^2^_0Mb_ = 0.328; r^2^_1Mb_ = 0.174; $\overline{{\mu }_{LD}}$ = 0.201) and SS (Supplementary Figure S6a). This difference was primarily driven by chromosomes 1, 4, 9, and 10, which had significant lower $\overline{{\mu }_{LD}}$ than IDT (Supplementary Figure S6b). Era-based observation of the evolution of LD demonstrates a subtle increase in LAN LD metrics (Δ(era 4-era 0): Δr^2^_0Mb_ =  + 0.042; Δr^2^_1Mb_ =  + 0.022; Δ $\overline{{\mu }_{LD}}$ =  + 0.047), significant only between era 3 and 4. On the opposite, IDT LD had decreasing LD indicators values, notably marked by a sharp significant decrease between era 1 and era 2 (Δ(era 4-era 0): Δr^2^_0Mb_ = -0.063; Δr^2^_1Mb_ = -0.066; Δ $\overline{{\mu }_{LD}}$ = -0.065). These two dynamics lead to a reversal in LD in era 4, with LAN having superior metrics values than IDT (Supplementary Figure S6c).

### Increase in population differentiation

We investigated genome-wide population differentiation using the Weir and Cockerham fixation index (F_ST_) to assess divergence between the two heterotic groups of the main heterotic pattern (SS and NSS) and between its ancestral subdivisions (SS, IDT, and LAN). The average F_ST_ between SS and NSS genotypes was 0.218, supporting strong differentiation between the two groups of the heterotic pattern. Comparison between chromosomes revealed significant differences in mean F_ST_ values, variance, and proportion of highly differentiated markers (i.e., F_ST_ > 0.75). Chromosome 10 had the lowest differentiation (F_ST_ = 0.156; var-F_ST_ = 0.027; P[F_ST_ > 0.75] = 0.002), whereas chromosome 6 displayed the highest (F_ST_ = 0.283; var-F_ST_ = 0.068; P[F_ST_ > 0.75] = 0.045) (Supplementary Table 2).

**Fig. 5:**
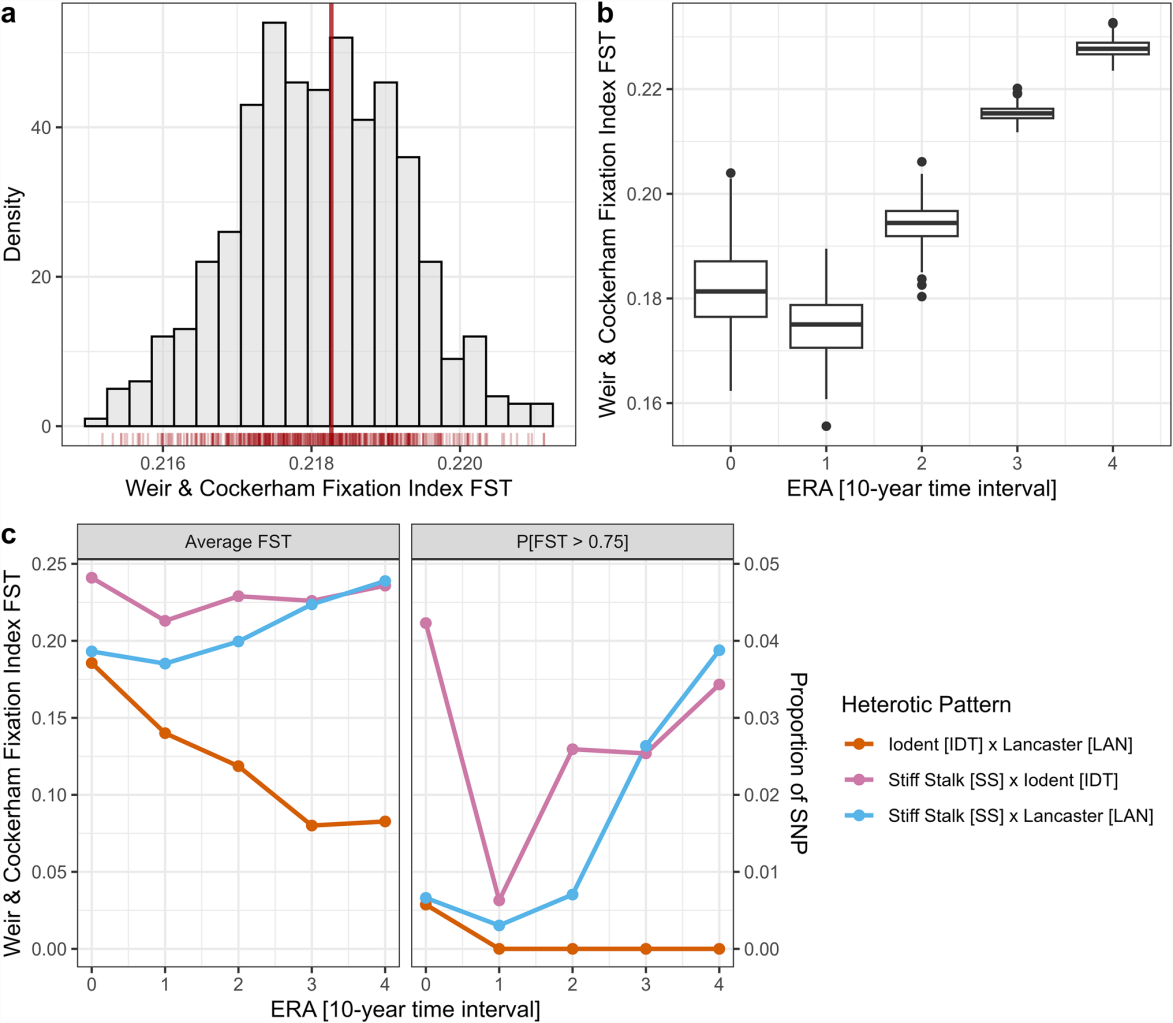
Differentiation between heterotic groups, measured by Weir & Cockerham fixation index FST, and its evolution across time **a** Bins-separated density chart of the bootstrapped average Weir & Cockerham fixation index FST for the pattern Stiff Stalk/Non-Stiff Stalk, all eras confounded. **b** Boxplot of the evolution of the bootstrapped Weir & Cockerham fixation index FST, between era 0 and era 4, for the pattern Stiff Stalk/Non-Stiff Stalk. **c** Evolution of the average Weir & Cockerham fixation index FST, between era 0 and era 4, for all pairwise ancestral heterotic groups (Stiff Stalk–Iodent–Lancaster) combinations (K = 3 configuration)

Genome-wide visualization of F_ST_ values revealed no large distinct hotspots of high differentiation (i.e., top 1% F_ST_ values; Supplementary Figure S7). Bootstrap analysis applied to F_ST_ computation (i.e., 200 repetitions with randomized 10% subsampling of inbreds from each group) showed normal distribution and low standard deviation, with an average of 0.218 ± 0.001 (Fig. 5a).

The division of NSS into its ancestral subgroups revealed a non-significant (p-value = 0.059) contrast in differentiation between SS and each subgroup. The F_ST_ values for SS-IDT and SS-LAN dataset pairs were 0.228 (P[F_ST_ > 0.75] = 0.027) and 0.225 (P[F_ST_ > 0.75] = 0.025), respectively. However, chromosome-specific evaluations of F_ST_ revealed significant differences for chromosomes 1, 2, and 4. Genome-wide patterns of F_ST_ showed similar shapes for the two comparisons, differing only in the scale of effects (Supplementary Figure S8). No subgroup-specific F_ST_ patterns were found. The IDT-LAN pair showed a low differentiation with an average genome-wide F_ST_ of 0.008 and an absence of high differentiation loci (max-F_ST_ = 0.52).

The evolution of the F_ST_ across eras revealed a significant increase in differentiation for the SS-NSS heterotic pattern. The value rose from 0.178 (var-F_ST_ = 0.032; P[F_ST_ > 0.75] = 0.003) in era 0 to 0.223 in era 4 (var-F_ST_ = 0.0513; P[F_ST_ > 0.75] = 0.026). Random sampling method on era-divided average F_ST_ showed reduced standard deviation and fewer outliers in recent eras (Fig. 5b). Within NSS, the evolution of differentiation between Iodent and Lancaster demonstrated a marked decline over time. In era 0, F_ST_(IDT-LAN) was comparable to the one between SS and LAN. By era 4, F_ST_(IDT-LAN) severely reduced, reaching close to null differentiation values (Fig. 5c). This dynamic supports the observed merging of these subgroups into NSS.

### Analysis of intragroup and intergroup genotype distances

Analysis of the Hamming distance between inbreds within each group showed a significant (*p*-value < 0.001) evolution, with an average decrease of 15% between era 0 and era 4. This deviation is particularly visible in the NSS, which went from an average of 0.379 in era 0 to an average of 0.318 in era 4 (a loss of 16.1%), compared to a loss of 1.4% in the SS (Table 3). Overall, pairwise Hamming distance calculations matched previously observed trends in MAF and F_ST_. On the contrary, calculation of pairwise between-group Hamming distance for the SS-NSS genotypes was not in accordance with the F_ST_ results and sustained a small decrease between era 0 and era 4, with a change of − 2.33% and an average distance of 0.419.

**Table 3:** Evolution of the Hamming distance between and inside the heterotic groups (mean ± SD)

ERA	BETWEEN SS-NSS	INTRA SS	INTRA NSS	BETWEEN IDT-LAN	INTRA IDT	INTRA LAN
0	0.430^a^ ± 0.025^b^	0.287 ± 0.087	0.379 ± 0.079	0.418 ± 0.027	0.246 ± 0.081	0.370 ± 0.079
1	0.425 ± 0.025	0.293 ± 0.070	0.374 ± 0.061	0.406 ± 0.033	0.292 ± 0.057	0.369 ± 0.055
2	0.423 ± 0.023	0.278 ± 0.061	0.359 ± 0.057	0.389 ± 0.035	0.293 ± 0.057	0.361 ± 0.049
3	0.416 ± 0.018	0.289 ± 0.055	0.319 ± 0.059	0.359 ± 0.035	0.274 ± 0.048	0.333 ± 0.051
4	0.420 ± 0.015	0.283 ± 0.061	0.318 ± 0.051	0.348 ± 0.031	0.282 ± 0.042	0.305 ± 0.053

^a^mean Hamming distance; ^b^standard deviation of the Hamming distance

## Discussion

In the present study, we assessed the genetic diversity and population structure of Limagrain mid-early/mid-late maize germplasm, using the entire inbred collection of 84,841 genotypes, analyzed on the 18 K Affymetrix SNP array.

### Population structure and breeding history of commercial European breeding program

The temporal genetic analysis, spanning the recorded history of this specific commercial European hybrid breeding program, enabled us to obtain a comprehensive overview of its evolution in genetic diversity and differentiation. Clustering of ancestral inbreds (i.e., era 0 to era 2 genotypes) suggests limited initial population structure, with three relatively close groups, i.e., Stiff Stalk, Iodent, and Lancaster (Mikel 2011; van Heerwaarden et al. 2012; Beckett et al. 2017). In contrast, era 3 and era 4 genotypes were structured into two clearly distinct clusters, consistent with the modern heterotic management practice of the Stiff Stalk and Non-Stiff Stalk heterotic pattern, described by Mikel (2008).

It has to be noted that these analyses are inherently influenced by the sample characteristics and marker quality of the dataset (Flint-Garcia et al. 2003; Mayer et al. 2017). The differences in sample size across heterotic groups and eras, particularly between the pre-GS period (eras 0–2) and the GS period (eras 3–4), may introduce bias in the diversity metrics’ computation. Prior to era 3, the genotypes included do not fully represent the genetic diversity of their respective eras, as they only reflect selected parental inbreds actively used in the current breeding program. Inbreds that did not contribute to modern schemes and provide descendants to eras 3 or 4 were not genotyped and were consequently absent from the dataset. As a result, we can assume that the true genetic diversity monitored during eras 0 to 2 may differ substantially from that captured in this study. This may lead to an overestimation of the metrics if non-genotyped materials of early eras consisted of large families of sister lines or closely related genotypes. Conversely, genetic diversity may have been underestimated if the missing genotypes represented sources of novel variation, such as contributions from additional landraces, ancestral populations, or other genetic backgrounds. The order of magnitude, direction, and global consequences of this potential bias remain uncertain due to a lack of concrete evidence.

Regarding markers, marker density and a possible ascertainment bias may have affected our results. We used in this study the Limagrain-designed 18 K SNP Chip, which was assembled based on the widely used Illumina 50 K SNP Chip. Since its creation, the 50 K SNP Chip has been extensively used in diversity analysis, notably to characterize landrace material, or other breeding programs (van Heerwaarden et al. 2012; Westengen et al. 2012; Schaefer and Bernardo 2013a; Zhang et al. 2018). The Illumina array includes around 30 K Panzea-based SNP, derived from diverse material, and around 11 K SNP, found by Syngenta, that discriminate B73 and MO17 inbred lines, the two main heterotic pool founders (Ganal et al. 2011). An ascertainment bias was consequently found for these Syngenta markers by Frascaroli et al. (2013). The Limagrain 18 K SNP Chip was mainly developed with the public Panzea markers, with only additional private SNPs, which supports low expected ascertainment bias. The superior proportion of intermediate-frequency SNPs, shown in the MAF distributions (Fig. 2), could underline a remaining ascertainment bias.

It is important to recognize that such commercial SNP arrays are primarily designed for high-throughput genotyping and optimized to maximize marker informativeness and capture broad polymorphism among elite breeding lines and across diverse selection programs. As a result, low-frequency variants are often deliberately excluded during SNP selection, leading to a genetic diversity profile that predominantly reflects intermediate-frequency alleles. While this does not affect the detection of major diversity patterns, it may limit the contribution of rare or lineage-specific variants from underutilized genetic backgrounds. Consequently, although the dataset provides robust insights into overall diversity trends, it may not fully capture the total genetic variation present. We acknowledge this as a limitation of the study and have accordingly tailored our interpretations to reflect germplasm-wide evolutionary patterns rather than rare-variant dynamics. In addition, as previously noted, although ascertainment bias could be impactful in our study, the SNPs included in our genotyping array were discovered using founder material and ancestral inbred lines. Since most of these inbreds are present in era 0, and the majority of subsequent lines (eras 1 to 3) are direct descendants of these founders, we do not anticipate a strong time-dependent evolution of ascertainment bias in our dataset.

Based on differentiation metrics results, we provided evidence that the increase in genetic divergence between the Stiff Stalk and Non-Stiff Stalk heterotic groups, that was first highlighted by Hallauer et al. (1988), was continuously reinforced over time with further selection. This evolution is the consequence of hybrid breeding schemes, and tester-based selection, like MRRS, which maximize heterotic pools’ separation (Hull 1945). The increasing proportion of fixed markers in each heterotic group, combined with an overall reduction in admixed genotypes, further endorses this observation (Technow et al. 2021). The increase in F_ST_ variance denotes higher variability in differentiation at the marker level, highlighting specific regions with more extreme values. Application of bootstrap methodology reveals a decreasing variance in bootstrapped average F_ST_. To mitigate the sampling bias, we only used inbreds (i.e., excluded selection lines) as they are more evenly distributed than the global dataset in the different eras. This analysis also reveals increasing within-group similarity in their differentiation response and possibly indicates lower residual admixture within genotypes.

Differentiation in early eras (era 0 to era 2) appears superior to other studies and may indicate differences between American and European founders of dent germplasm (van Heerwaarden et al. 2012; Romay et al. 2013; Beckett et al. 2017). We suggest that this contrast derives from the specific origin of European dent material. European dent breeding programs, initiated post-World War II, relied on a narrow subset of high-yielding elite material of that time (inbreds and hybrids), with a stringent selection for earliness, adaptation to cooler growing conditions, and resistance to higher local disease pressure, leading to a strong bottleneck (Messmer et al. 1992; Reif et al. 2005; Tenaillon and Charcosset 2011). Consequently, first- and second-cycle European dent inbreds trace back to fewer elite progenitors than US germplasm, with specific US backgrounds missing in European germplasm (e.g., Krug, Osterland, and other southern dents). Furthermore, for this period of introduction, these US lines had an already well-established heterotic structure, which further diminished intragroup genetic diversity and increased between-group differentiation (Smith et al. 2006, 2022).

Comparison of F_ST_ estimates and their evolution confirms the high differentiation between the two dent heterotic groups in modern material (era 4: F_ST_ (SS-NSS) = 0.22). Our results reach differentiation levels higher than (F_ST_ = 0.18) those observed for a flint-dent heterotic pattern within an ongoing early maize hybrid breeding program (Allier et al. 2019). Compared to the dent-dent heterotic pattern, the flint-dent heterotic pattern divergence can be explained by their historic geographical separation and initial adaptation to different environments (see introduction and Unterseer et al. 2016). The historical aspect of this genetic differentiation renders the flint-dent heterotic pattern as a strong divergent population structure, which has been extensively studied (Messmer et al. 1992; Camus-Kulandaivelu et al. 2006; Rincent et al. 2014; Gouesnard et al. 2017). Patterns of increasing differentiation over time have been documented across other heterotic groups and breeding programs (Van Inghelandt et al. (2010); van Heerwaarden et al. (2012)). Such evolution is supported by theoretical studies which reported that increasing divergence is correlated with increased heterosis (Legarra et al. 2023). The increasing F_ST_ values within maize hybrid breeding programs highlight the long-term commitment to increasing heterotic pools divergence. In comparison to other hybrid crops, where genetic differentiation between groups is also of key interest, inbred sorghum and rye lines exhibited similar F_ST_ values between parental groups than the one observed in our study, with estimates around 0.25 and 0.23, respectively (Mindaye et al. 2015; Bauer et al. 2017).

### Evolution of Non-stiff Stalk reveals a genetic homogenization phenomenon

**Fig. 6:**
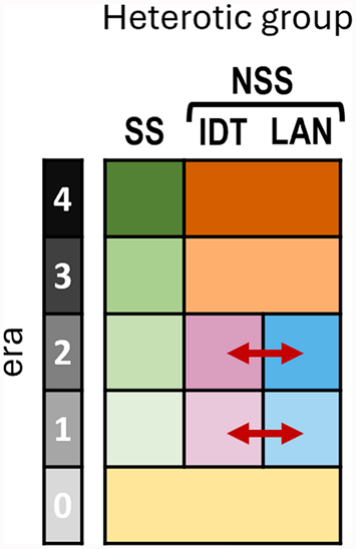
Graphical representation of the population structure genetic homogenization event. Red arrows represent material transfer

Additional effects could explain the sharp evolution of intragroup and intergroup metrics, impacting particularly the Non-Stiff Stalks, between era 2 and era 3. Analysis of population structure and clustering reveals the convergence of the initial Non-Stiff Stalk ancestral Iodent and Lancaster clusters into a new modern meta-heterotic group (Fig. 6). This mechanism is comparable to the phenomenon described as “genetic homogenization” in animal population genetics studies, notably Rhymer and Simberloff (1996), Olden et al. (2004), and Baggio et al. (2018). These studies reported the development of a single, large population, from originally distinct populations, and its impacts on genetic diversity and differentiation, comparable to what we observe in our study.

The observed genetic homogenization of the Iodent and Lancaster heterotic groups into the Non-Stiff Stalk likely resulted from multiple demographic factors and intentional breeding strategies. Using our findings and previous studies, primarily those related to maize dent germplasm (Romero-Severson et al. 2001; Smith et al. 2004; Mikel and Dudley 2006; White et al. 2020), we propose the following step-by-step process which led to the observe population structure: (1) founder effects within groups, (2) gene flow between groups, and (3) unrestrained admixture.

First, a significant factor contributing to genetic homogenization could be founder effects within both groups (Olden et al. 2004). The Iodent germplasm is based on a single early-maturing strain of selected Reid material (“[Idt]4A”). Later-developed Iodent material was produced by the recurrent selection of 8 founders chosen for their earliness and yield (Troyer 1999, 2004; Tracy and Chandler 2006). These conditions led to a narrow founding base with few initial progenitors (Mikel and Dudley 2006; Mikel 2011). Access to such novel germplasm by seed companies relied on the selfing of 1980s’ and 1990s’ commercial hybrids in the USA and in Europe. While these hybrids were genetically very similar, they deeply modified European maize practices and were represented on average in 20% of available commercial material pedigree (Darrah and Zuber 1986; Barrière et al. 2006; Smith et al. 2006; White et al. 2020). The narrow founder population size of Iodent material is demonstrated in our study by the observed lowest average MAF in era 0. In contrast to the USA, the use of Lancaster germplasm in Europe was constrained by difficulties related to its late maturity. This stringent selection bottleneck of material suited for cold environments reduced the pool of potential Lancaster founders and discarded other origins (e.g., Southern Dent). Consequently, both Iodent and Lancaster European clusters were founded on narrower genetic bases than American ones, potentially predisposing them to genetic homogenization.

Second, gene flow between the two ancestral heterotic groups may have been employed as a solution to address breeding challenges (Rhymer and Simberloff 1996). The limited founding population of Iodent likely led to the introduction of other genetic backgrounds to enhance diversity, in part through the use of commercial hybrids as progenitors. This occurred notably with Lancaster-types leading to the “non-canonical subgroup Iodent-Oh43 non-stiff stalk” (White et al. 2020). Lancaster traits have been incorporated into Iodent germplasm to improve resilience and performance under diverse environmental conditions, by adding variability to the narrow Iodent genetic base (White et al. 2020). Breeders also appear to have intentionally introduced Iodent traits to enhance early maturity and stability in Lancaster germplasm, ensuring its adaptability to European colder climates (Barrière et al. 2006). This gene flow can be demonstrated by the continuous significant decrease in pairwise F_ST_ between Iodent and Lancaster, from era 0 to era 4. These consequences hint for an important transfer of genetic material (e.g., migration and/or admixture) between the two groups (Olden et al. 2004).

Finally, full unrestrained admixture of the two groups was achieved in era 3, with the implementation of a global genomic selection calibration pipeline for the Stiff Stalk–Non-Stiff Stalk heterotic pattern, thus removing boundaries between Iodent and Lancaster. This breeding choice was made to simplify heterotic pattern management (e.g., number of testers, number of possible crosses) and streamline the incorporation of novel germplasm (i.e., either SS or NSS). This strategy promoted the convergence of genetic profiles, contributing to the observed loss of clear heterotic boundaries, the gradient-like separation in the PCA, and the overt decrease in genetic diversity (decrease of − 27.75% of average MAF) between era 2 and era 3, as observed in the genetic homogenization cases mentioned above (Rhymer and Simberloff 1996).

These combined factors suggest a complex interplay of founder effects, migration, admixture, and artificial selection based on tester evaluation in driving the observed structure changes. The role of each factor (e.g., genetic drift, gene flow, selection, admixture) in influencing differentiation and its evolution depends on the selection pressure and the effective population size (Baggio et al. 2018). Previous studies reported the small effective population size of commercial maize breeding, influenced by the repetitive use of key elite materials, the genetic proximity between its founders, and the multiple genetic bottlenecks (e.g., founders’ effects and precocity bottleneck between US and European dent germplasm) (Guzman and Lamkey 2000; Romero-Severson et al. 2001; White et al. 2020). In our case with high artificial selection pressure and low effective population size, disentangling drift and selection can be challenging, a situation termed High Drift-High Selection regime (Lamkey and Lorenz 2014; Gerke et al. 2015; Desbiez-Piat et al. 2021). Without considering phenotypic information, we can only speculate that observed increased divergence between heterotic groups, seen with an increased average F_ST_, could originate from the effects of selection for beneficial alleles in a divergent population structure and from heterotic group-specific genetic hitchhiking (Smith and Haigh 1974; Allier et al. 2019; Civan et al. 2024). This assumption could be supported by the significant increase in intragroup fixity, which likely originates from the stacking of beneficial alleles from elite inbred recycling in hybrid recurrent reciprocal selection. This will be addressed in a future study.

It should, however, be noted that, despite the observed significant reduction in diversity present in the Non-Stiff Stalks in previous eras, current levels of genetic diversity are comparable to that of the Stiff Stalk group, which remained relatively stable. These changes are thus not critical and only suggest that increased caution in diversity management is now needed within the breeding pipeline (e.g., inbred relatedness, tester use). Integration and use of new tools or methods, like Optimal Contribution Selection (OCS), or genetic resources bridging could be of interest (Simmonds 1993; Meuwissen 1997; Daetwyler et al. 2015; Sanchez et al. 2024). Further analyses of this genetic architecture evolution and attempts to separate selection-related effects from stochastic-linked demographic processes (e.g., genetic drift) are necessary to fully understand breeding-induced changes in genetic architecture and the impacts of the genetic homogenization demographic event.

### Application of large-scale time series analysis to breeding and estimation of genetic diversity

Our analysis identified a significant loss of allelic diversity within the global germplasm, predominantly driven by the Non-Stiff Stalk homogenization event and the progressive divergence between heterotic groups (SS and NSS). The application of large-scale temporal genotypic data enabled the monitoring of key genetic metrics and the identification of both global demographic processes and localized patterns of genetic changes. The use and extension of the indicator proposed by Technow et al. (2021), incorporating MAF checks at varying thresholds, provided additional insight into the interpretation of the evolution of marker-based genetic analyses. It allowed highlighting fixation dynamics not observable with mean MAF or He. This study thus serves as a further proof-of-concept for the application of these indicators and the research of new metrics in genetic analyses, reinforcing their utility in advancing diversity management in breeding schemes.

The analysis of allelic diversity during era 0 exposed significant differences in starting available genetic diversity between heterotic groups. The Stiff Stalks exhibited initially lower average MAF, higher proportion of fixed markers, and consistently higher LD across all marker distances compared to Non-Stiff Stalks. Elevated LD along the genome indicates reduced recombination events between markers, leading to the non-random association of alleles that segregate together during meiosis. This results in the creation of longer, less diverse haplotypes (Li et al. 2017; Bornowski et al. 2021). These contrasts abide by the historically reported narrower genetic base and smaller effective population size of Stiff Stalk germplasm, primarily derived from the Iowa Stiff Stalk Synthetic population and its three key elite founders (i.e., B14, B37, B73) (Gracen 1986; Troyer 1999; Romay et al. 2013).

Segmentation of the Non-Stiff Stalk reveals that the Iodent ancestral heterotic group had a significantly lower average MAF than Stiff Stalks. This observation supports the Iodent historical origin, with its even smaller effective population size and unique founder (White et al. 2020). Despite this reported distinctive source, additional genetic indicators (e.g., LD, MAF checks) did not match with this information, with Iodent in era 0 having similar MAF-based results and lower far-reaching LD compared to Stiff Stalks. These findings may suggest that the Iodent inbreds composing era 0 were already admixed with other sources, supporting the later introduction of already selected dent types in Europe, though the use of commercial hybrids (White et al. 2020; Coffman et al. 2020).

Observed regions of low diversity within SS population and their evolution across eras are consistent with previous studies of Stiff Stalk germplasm. Notably, the centromeric pattern of chromosome 2 and the starting telomeric region of chromosome 6 exhibit pronounced fixation, as documented by Allier et al. (2019) and White et al. (2020). Bornowski et al. (2021) further confirmed the fixation across these genomic regions, with the ascertainment of non-recombined B73 haplotypes, derived from C.I.540 and Tr9-1-1-6 founding populations, within commercial germplasm. Incorporating additional diversity into a divergent population structure has proven complex and risks negatively impacting differentiation. As a result, efforts to integrate new genetic material into the female pool have been limited, potentially explaining the long-term conservation of large fixed genomic fragments (Reif et al. 2005; Dempewolf et al. 2023).

Overall, while SNP-based analyses provide valuable insights into breeding programs’ evolution, additional methodologies could confirm and enhance these findings. Whole-genome sequencing or other advanced genotyping technologies could further validate our results (Romay et al. 2013; Gouesnard et al. 2017), and haplotype-based methods, which are closely linked to genotype similarity, identity-by-descent, and fragments transfer from founders’ populations, might refine the used indicators (Romero-Severson et al. 2001; Coffman et al. 2020). Such approaches would provide additional understanding of germplasm dynamics and inform strategies for integrating new material into breeding schemes.

## Conclusion

We presented a comprehensive description of the evolution of genetic diversity, differentiation, and population structure parameters in a commercial European hybrid dent maize breeding program. The availability of large-scale temporal genotypic data was utilized to assess the continuous evolution of multiple key genetic metrics. The results of our study indicated that current and previous inbred breeding schemes have led to a significant genome-wide increase in differentiation between Stiff Stalk and Non-Stiff Stalk heterotic groups. At the same time, diversity was strongly reduced, impacting primarily the Non-Stiff Stalks, which underwent genetic homogenization through population merging of ancestral Iodent and Lancaster groups. This event led to a major drop in allelic diversity and a significant increase in linkage disequilibrium in two decades, leading it now to similar levels as the Stiff Stalk heterotic group. These observations, while not critical for the moment, suggest that caution is now needed in the inbred development pipeline and tools for improved diversity management should be considered. This study highlights the critical impact of population structure management on between-group differentiation and its effects on intragroup diversity.

## Supplementary Information

Below is the link to the electronic supplementary material.

Supplementary file1 (PDF 2356 kb)

## Data Availability

The data analyzed in this study are part of an active commercial maize breeding program of Limagrain Europe and are composed of the complete genotypic history for its mid-early to mid-late maturity, from its founders to current experimental selection lines. As such, the data and genotypic material are confidential and protected as intellectual property or as trade secrets. Consequently, datasets are not publicly available. R code can be made available upon request.
