# Supplementary material for: Evolution of population structure in a commercial European hybrid dent maize breeding program and consequences on genetic diversity
Source: Theor Appl Genet. 2025 Aug 30;138(9):233. doi: 10.1007/s00122-025-05008-5 (PMC12397178; doi:10.1007/s00122-025-05008-5)
Supplement: Supplementary file 1 — Supplementary file1 (PDF 2356 kb) [file 122_2025_5008_MOESM1_ESM.pdf]

TITLE

Evolution of population structure in a commercial European hybrid dent maize breeding program and consequences on genetic diversity.

Short Title: European Dent Maize Diversity Evolution

AUTHORS

Romain Kadoumi (https://orcid.org/0009-0007-0560-4945)[1,2], Nicolas Heslot[1], Fabienne Henriot[1], Alain Murigneux[1], Mathilde Berton[1], Laurence Moreau (https://orcid.org/0000-0002-7195-1327)[2], Alain Charcosset (https://orcid.org/0000-0001-6125-503X)[2]

[1] Limagrain Field Seeds, 28 Route d'Ennezat, 63720 Chappes, France

[2] Génétique Quantitative et Evolution – Le Moulon, Institut National de la Recherche Agronomique et Environnementale, Université Paris-Sud, AgroParisTech, Université Paris-Saclay, F-91190 Gif-sur-Yvette, France

Corresponding author

Alain Charcosset (alain.charcosset@inrae.fr - https://orcid.org/0000-0001-6125-503X)

KEY MESSAGE

Differentiation between Stiff Stalk and Non-Stiff Stalk heterotic groups increased significantly over time, while genetic diversity within both groups declined, highlighting the impact of long-term selection in hybrid maize breeding.

KEY WORDS

Maize, Heterotic groups, population structure, diversity, differentiation, breeding

ACKNOWLEDGEMENT

This research was funded by *Limagrain Europe* and the *Agence Nationale de la Recherche et de la Technologie (ANRT*) *[CIFRE Grant N°2023/1777]* for RK. The authors would like to thank *Limagrain Field Seeds* for providing the molecular and pedigree data. The author RK wishes to thank everyone involved in the proofreading of this manuscript and the discussions around this project.

This file contains:

Supplemental Figure Legends

Supplemental Figures

Supplemental Table Legends

Supplemental Table

SUPPLEMENTARY TABLE LEGENDS

Supplementary Table 1 Evolution of the minor allele frequency [MAF global germplasm and for each heterotic group, with the decomposition of the Non-Stiff Stalk into its subclusters (mean ± SD)

Supplementary Table 2 Description of the FST across chromosomes for the heterotic pattern Stiff Stalk / Non-Stiff Stalk

SUPPLEMENTARY FIGURE LEGENDS

Supplementary Figure S1 Marker density of Limagrain Field Seeds proprietary 18K SNP Affymetrix genotyping array

Supplementary Figure S2 Evidence for the reduction in admixed genotypes. Evolution in the proportion of genotypes with CMAP strictly lower than 75% between era 0 and era 4. Heterotic groups are shown by distinct colors (SS in green – NSS in red).

Supplementary Figure S3 Evolution of Expected Heterozygosity He along the genome, in 1-Mb bins, for global germplasm

Supplementary Figure S4 Evolution of the Minor Allele Frequency [MAF] in the two heterotic groups (Stiff Stalk [SS] and Non-Stiff Stalk [NSS]), along the genome, in 2 Mb bins, between ERA 0 [pre-1990 germplasm] and ERA 4 [2020 germplasm]

Supplementary Figure S5 Evolution of the Minor Allele Frequency [MAF] in the two sub-heterotic groups of the Non-Stiff Stalk (Iodent [IDT] and Lancaster [LAN]), following K=3 clustering, along the genome in 2 Mb bins, between ERA 0 [pre-1990 germplasm] and ERA 4 [2020 germplasm].

Supplementary Figure S6 Analysis of Linkage Disequilibrium (LD) and its evolution between the three ancestral heterotic groups. Obtained results are measured by marker pairwise correlation (r^2). Heterotic groups are shown by distinct colors (a) Line plot of the LD according to the physical distance (in kb) between markers. (b) Dot plot of the average LD within 1 Mb of marker physical distance, for each chromosome (c) Evolution of LD based on three indicators (Average LD within 1-Mb; LD at 0-Mb; LD at 1-Mb)

Supplementary Figure S7 Evolution of Weir & Cockerham FST along the genome, in 1-Mb bins, for the Stiff Stalk/Non-Stiff Stalk heterotic pattern

Supplementary Figure S8 Evolution of Weir & Cockerham FST along the genome, in 1-Mb bins, for the each pairwise pattern using K = 3 clustering).

Supplementary Figure S9-13 Population Structure of the dataset according to ADMIXTURE origin assignation for K = 3. Barplots are realized per era and only computed for inbreds.

SUPPLEMENTARY TABLE

***Supplementary Table 1** Evolution of the minor allele frequency [MAF global germplasm and for each heterotic group, with the decomposition of the Non-Stiff Stalk into its subclusters (mean ± SD)*

	Global germplasm	***Stiff stalk***	***Non-stiff stalk***		
			Merged	*Iodent*	*Lancaster*
ERA 0	0.294[a] ± 0.128[b]	0.207 ± 0.159	0.284 ± 0.134	0.150 ± 0.100	0.274 ± 0.138
ERA 1	0.290 ± 0.130	0.206 ± 0.148	0.279 ± 0.136	0.193 ± 0.119	0.272 ± 0.135
ERA 2	0.284 ± 0.134	0.197 ± 0.150	0.267 ± 0.139	0.201 ± 0.134	0.266 ± 0.137
ERA 3	0.265 ± 0.144	0.214 ± 0.152	0.209 ± 0.147	0.191 ± 0.145	0.225 ± 0.152
ERA 4	0.263 ± 0.146	0.208 ± 0.155	0.200 ± 0.143	0.188 ± 0.142	0.232 ± 0.150

a: mean MAF ; b: standard deviation of the MAF

***Supplementary Table 2** Description of the FST across chromosomes for the heterotic pattern Stiff Stalk / Non-Stiff Stalk*

	**AVERAGE FST**	**VARIANCE FST**	**P[FST > 0.75]**
Chromosome 1	0.236	0.052	0.027
Chromosome 2	0.240	0.049	0.022
Chromosome 3	0.196	0.041	0.010
Chromosome 4	0.220	0.045	0.005
Chromosome 5	0.172	0.032	0.003
Chromosome 6	0.283	0.068	0.045
Chromosome 7	0.234	0.057	0.045
Chromosome 8	0.205	0.048	0.016
Chromosome 9	0.229	0.050	0.023
Chromosome 10	0.157	0.027	0.002

SUPPLEMENTARY FIGURES

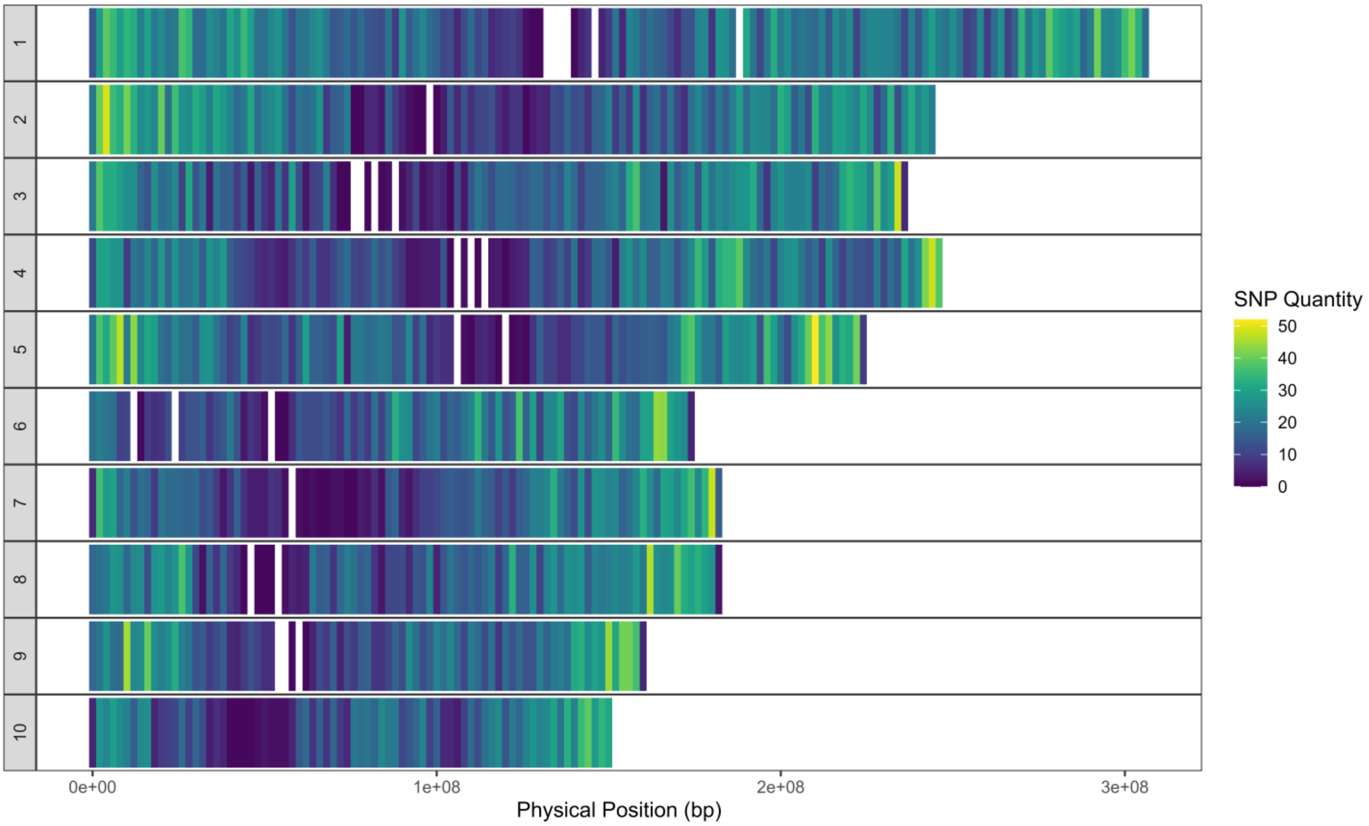

Supplementary Figure S1 *Marker density of Limagrain Field Seeds proprietary 18K SNP Affymetrix genotyping array*

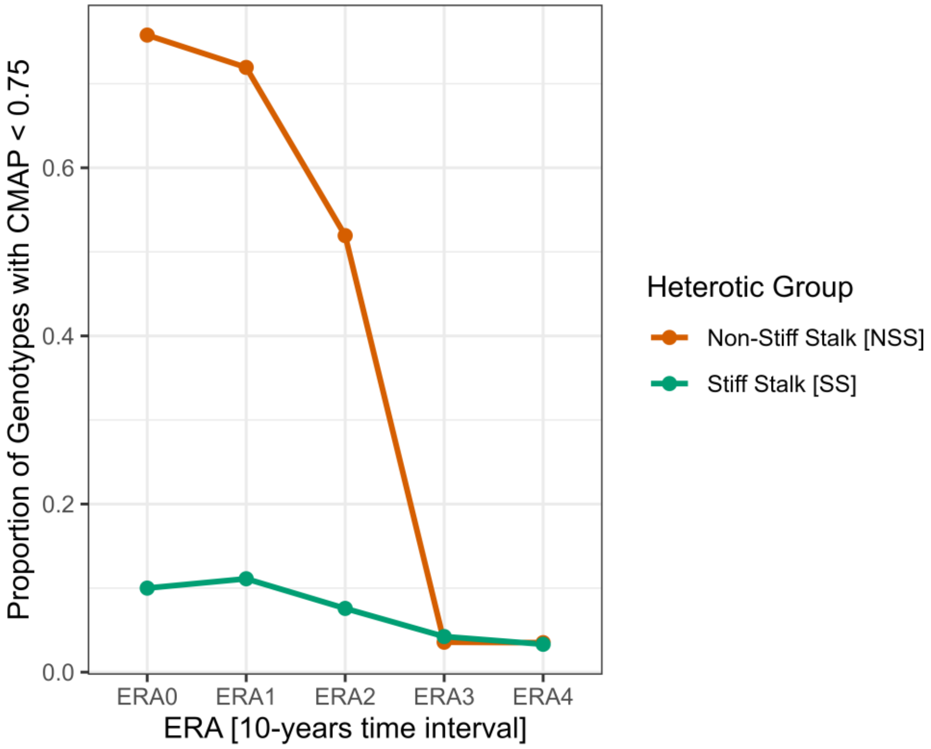

Supplementary Figure S2 *Evidence for the reduction in admixed genotypes. Evolution in the proportion of genotypes with CMAP strictly lower than 75% between era 0 and era 4. Heterotic groups are shown by distinct colors (SS in green – NSS in red).*

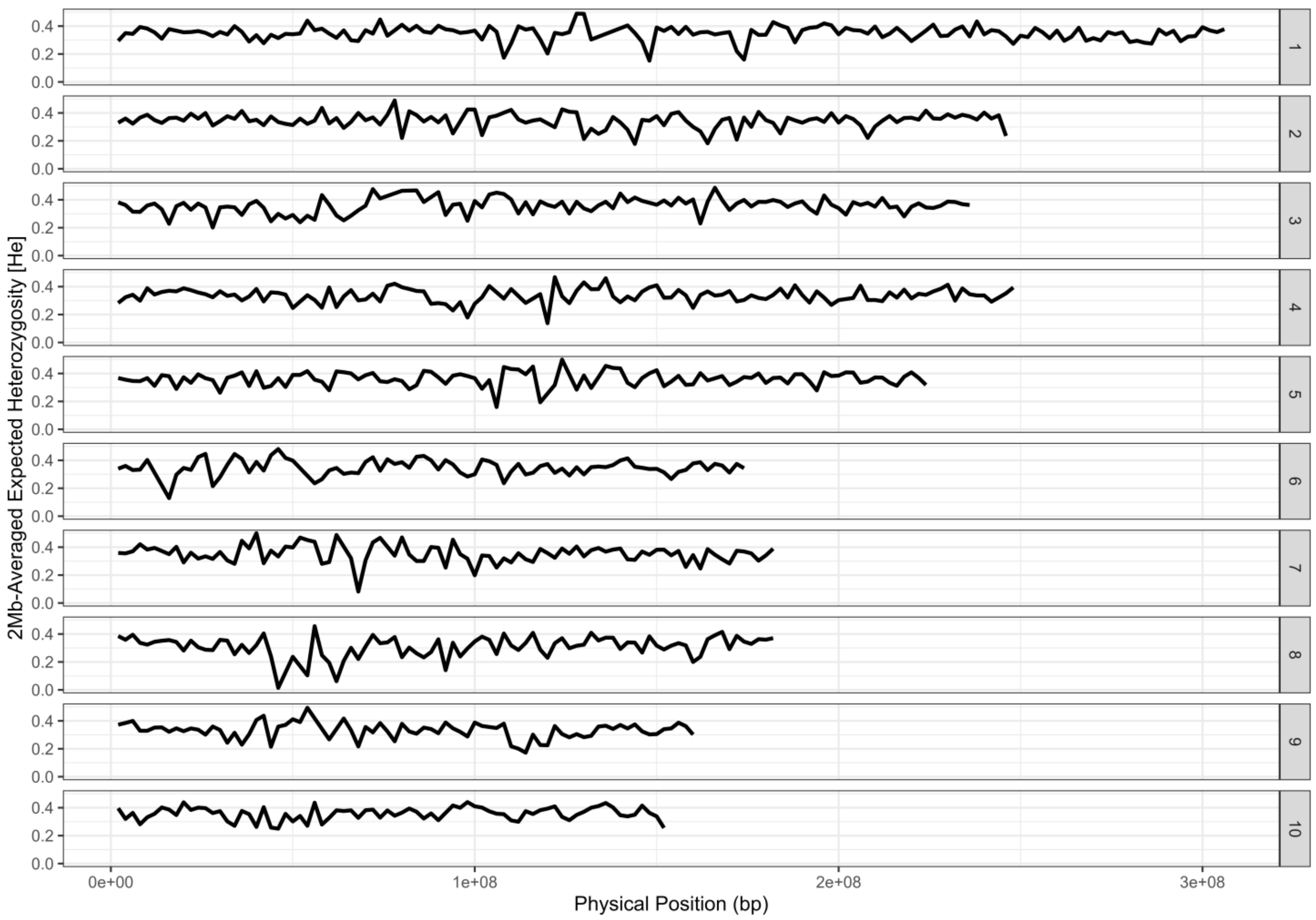

Supplementary Figure S3 *Evolution of Expected Heterozygosity He along the genome, in 1-Mb bins, for global germplasm*

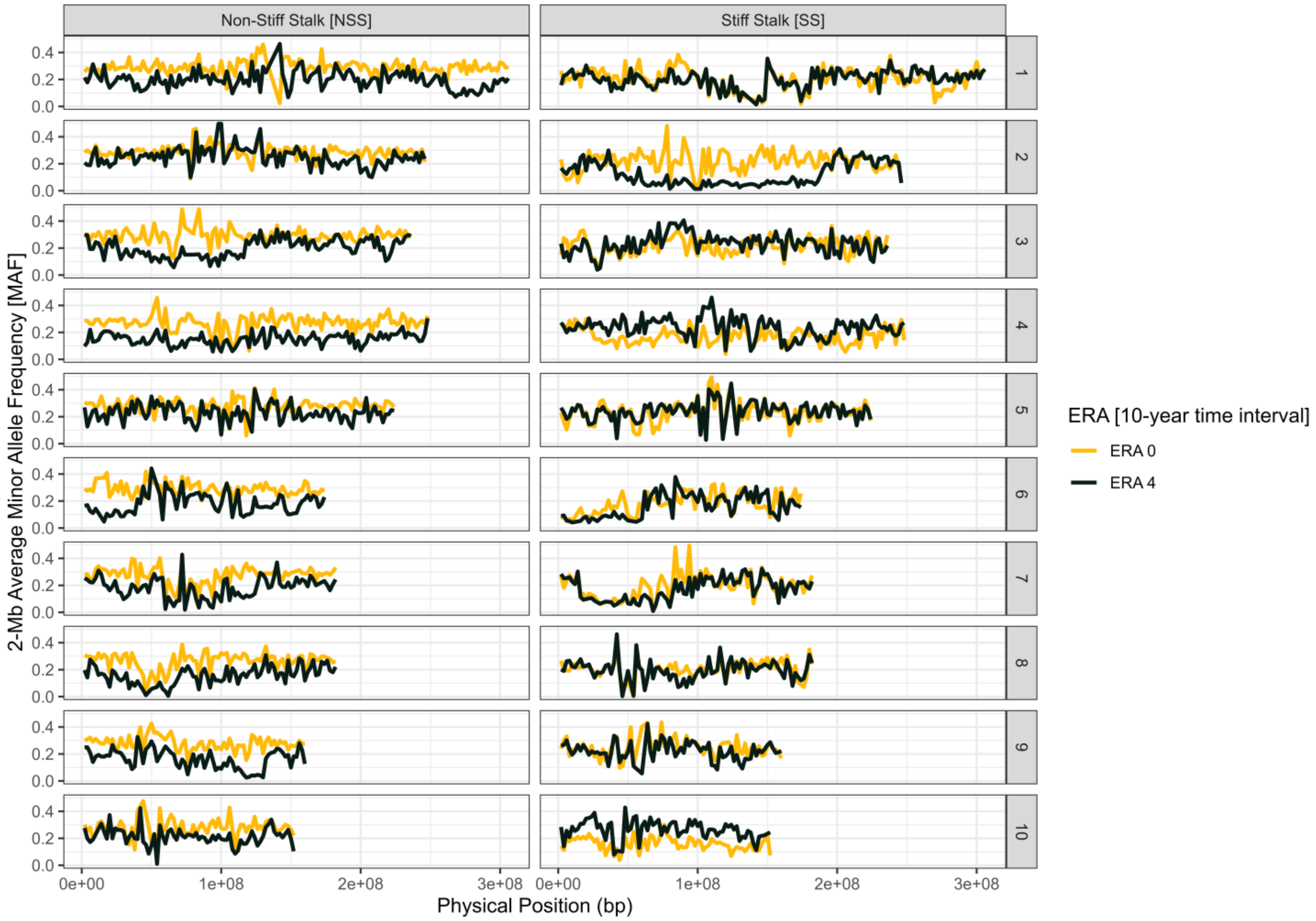

Supplementary Figure S4 *Evolution of the Minor Allele Frequency [MAF] in the two heterotic groups (Stiff Stalk [SS] and Non-Stiff Stalk [NSS]), along the genome, in 2 Mb bins, between ERA 0 [pre-1990 germplasm] and ERA 4 [2020 germplasm]*

88

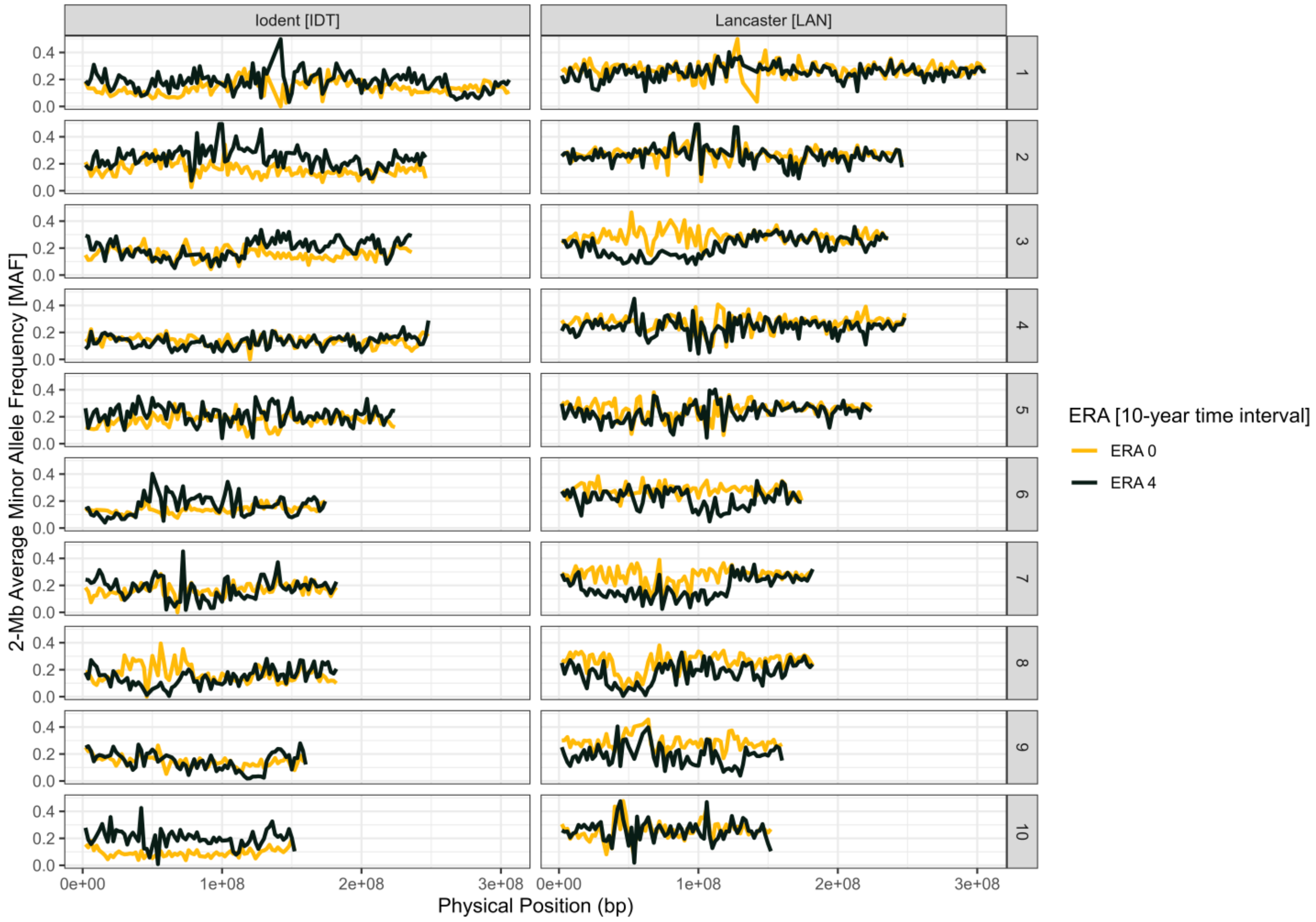

89 ***Supplementary Figure S5*** *Evolution of the Minor Allele Frequency [MAF] in the two sub-heterotic groups of the Non-Stiff Stalk (Iodent [IDT] and Lancaster [LAN]), following K=3 clustering, along the genome in 2 Mb bins, between ERA 0 [pre-1990 germplasm] and ERA 4 [2020 germplasm].*

90

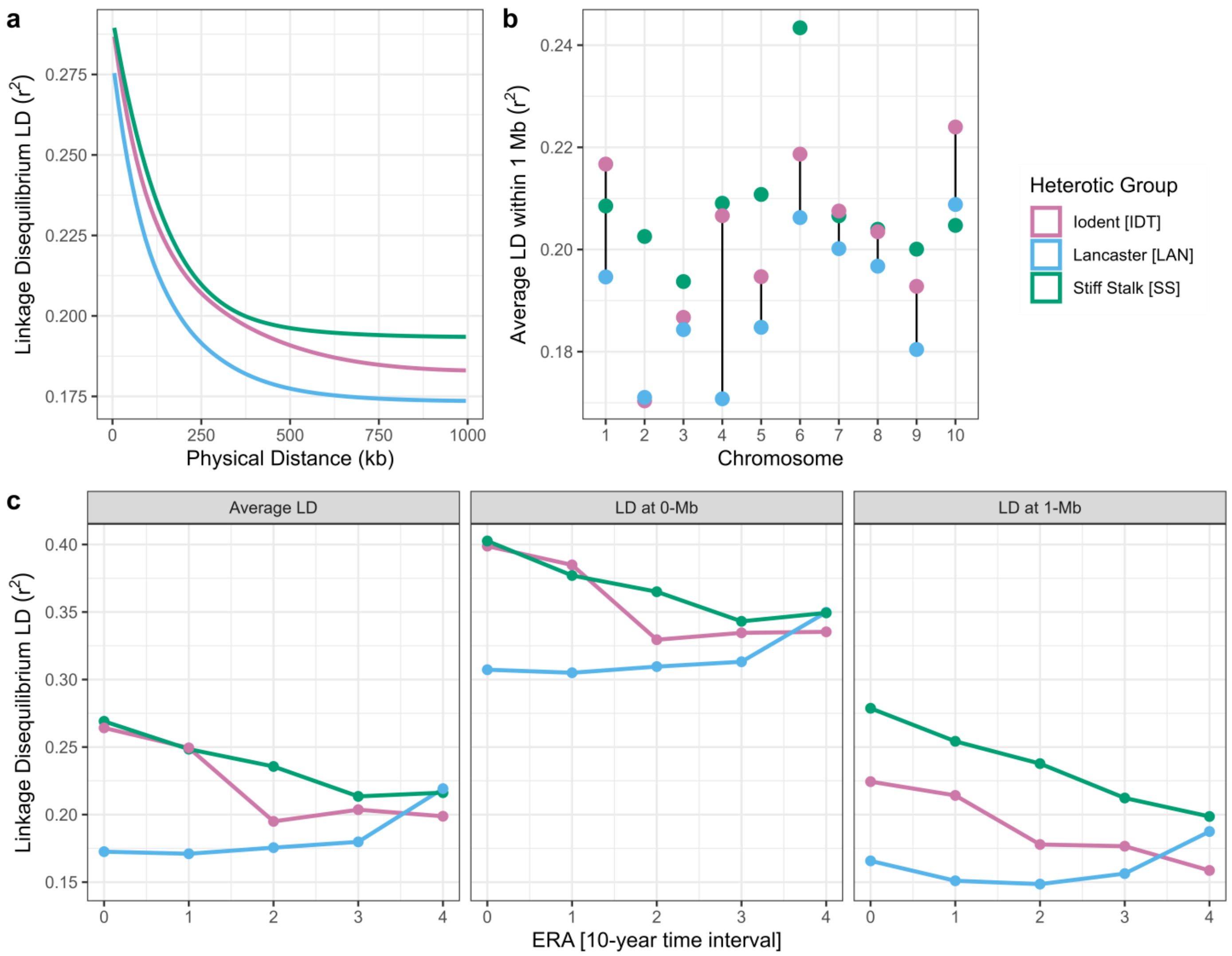

Supplementary Figure S6 *Analysis of Linkage Disequilibrium (LD) and its evolution between the three ancestral heterotic groups. Obtained results are measured by marker pairwise correlation (r^2). Heterotic groups are shown by distinct colors (a) Line plot of the LD according to the physical distance (in kb) between markers. (b) Dot plot of the average LD within 1 Mb of marker physical distance, for each chromosome (c) Evolution of LD based on three indicators (Average LD within 1-Mb; LD at 0-Mb; LD at 1-Mb)*

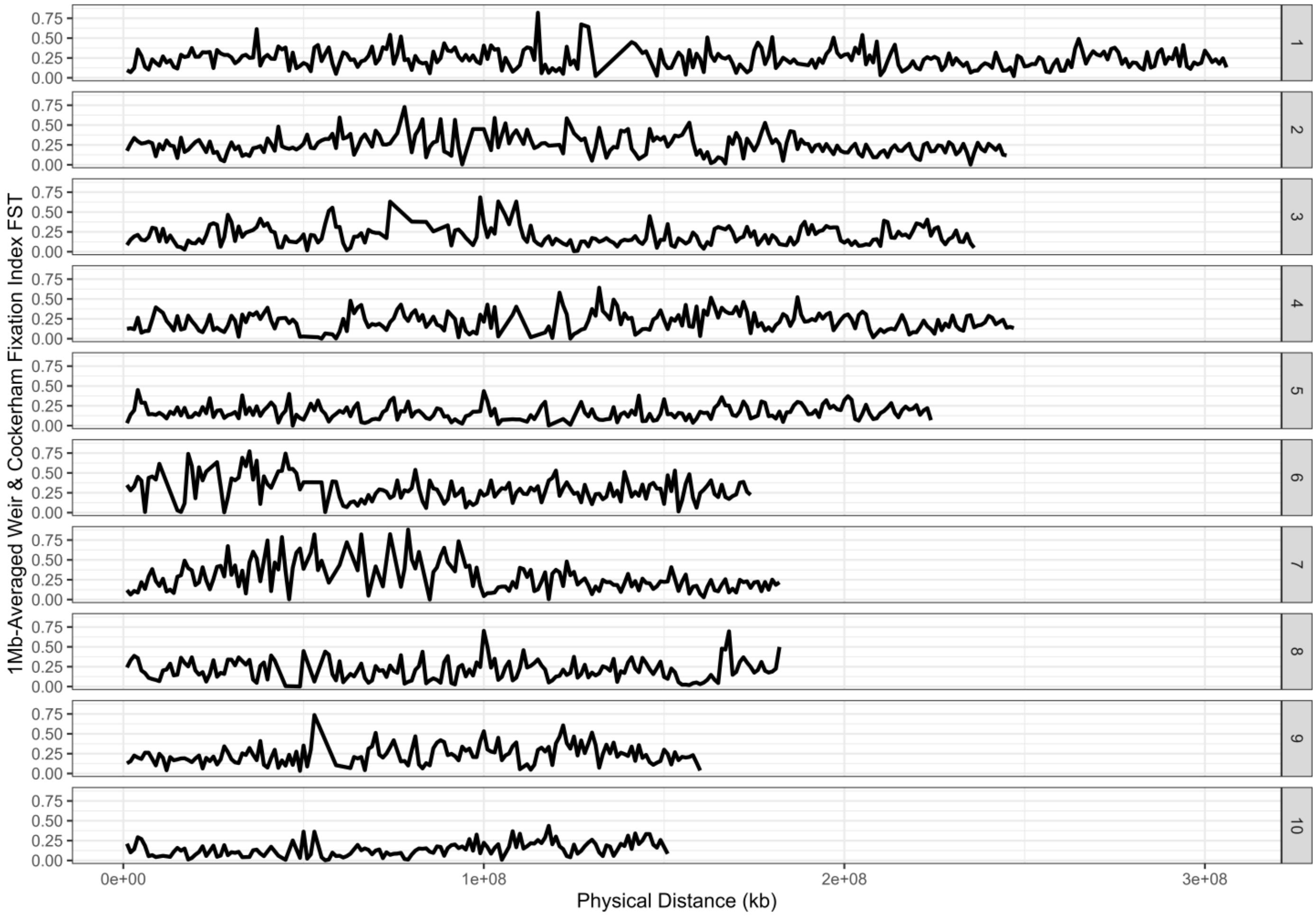

Supplementary Figure S7 *Evolution of Weir & Cockerham FST along the genome, in 1-Mb bins, for the Stiff Stalk/Non-Stiff Stalk heterotic pattern*

93

94

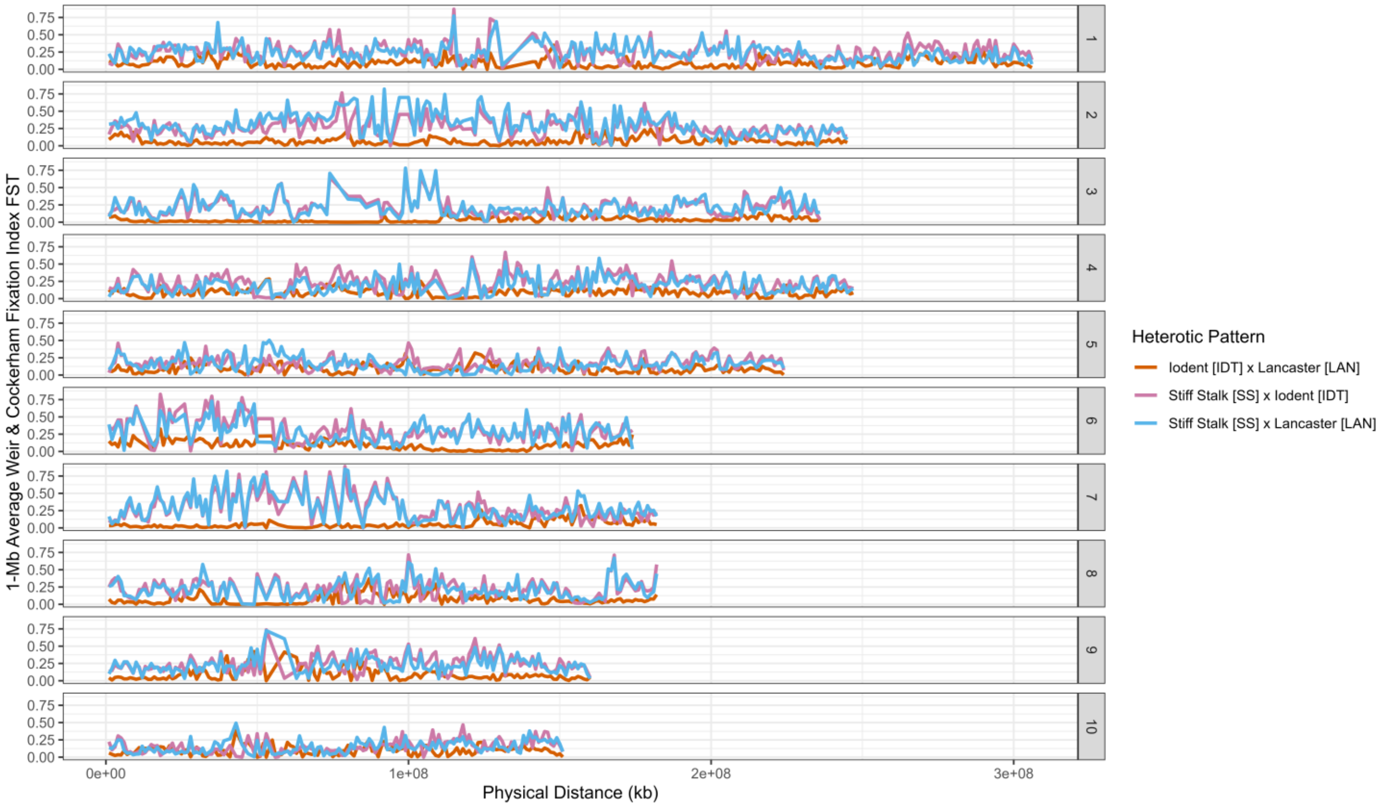

Supplementary Figure S8 *Evolution of Weir & Cockerham FST along the genome, in 1-Mb bins, for the each pairwise pattern using K = 3 clustering).*

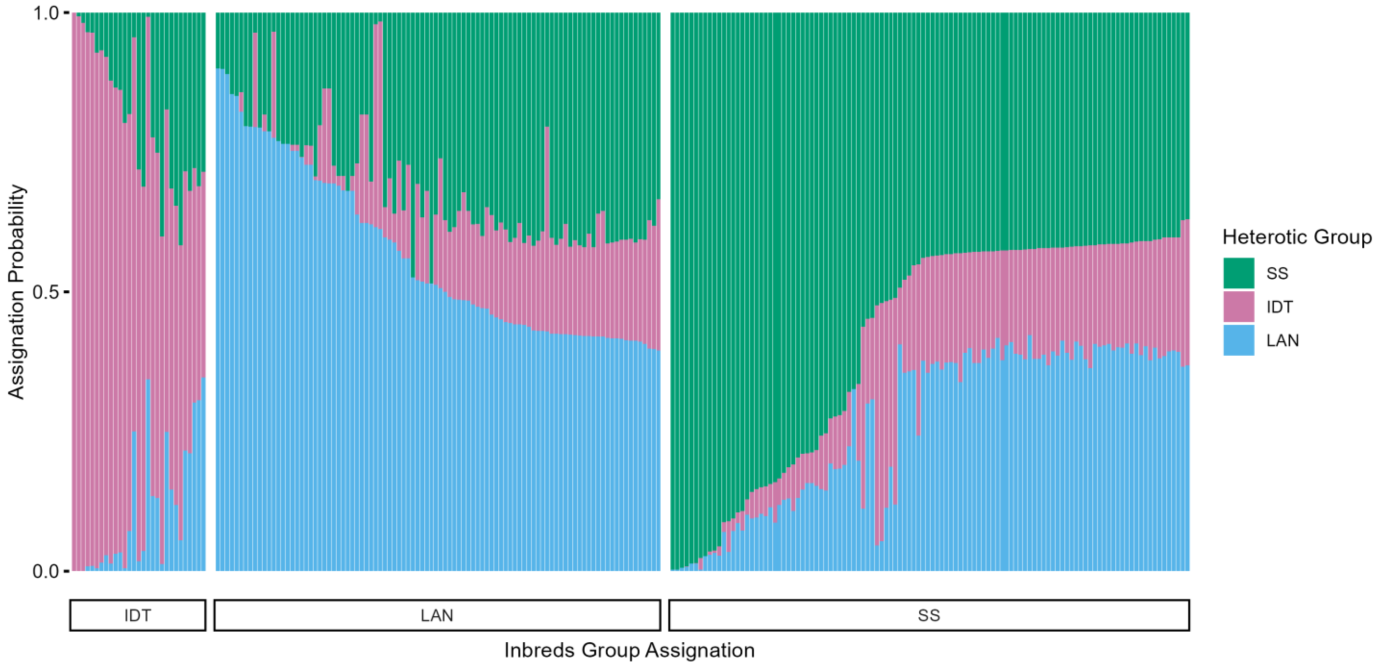

Supplementary Figure 9 *Population Structure of the dataset according to ADMIXTURE origin assignation for K = 3 and for era 0.*

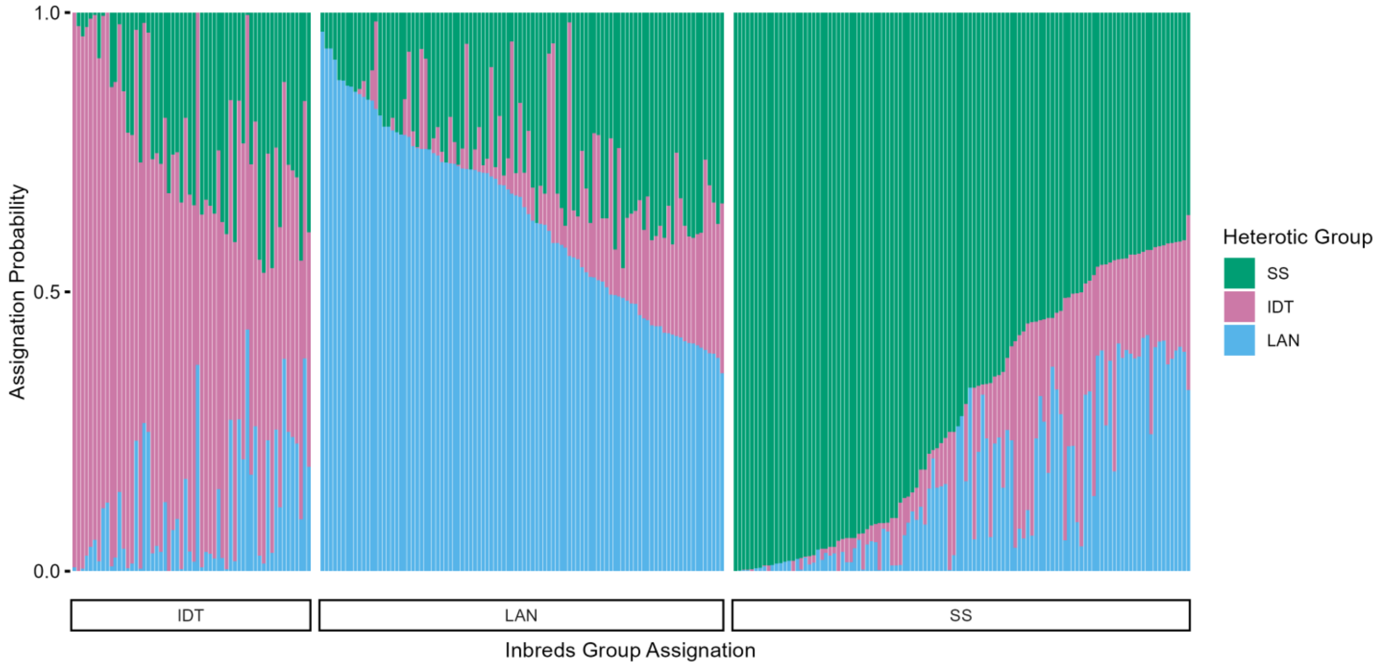

Supplementary Figure 10 *Population Structure of the dataset according to ADMIXTURE origin assignation for K = 3 and for era 1.*

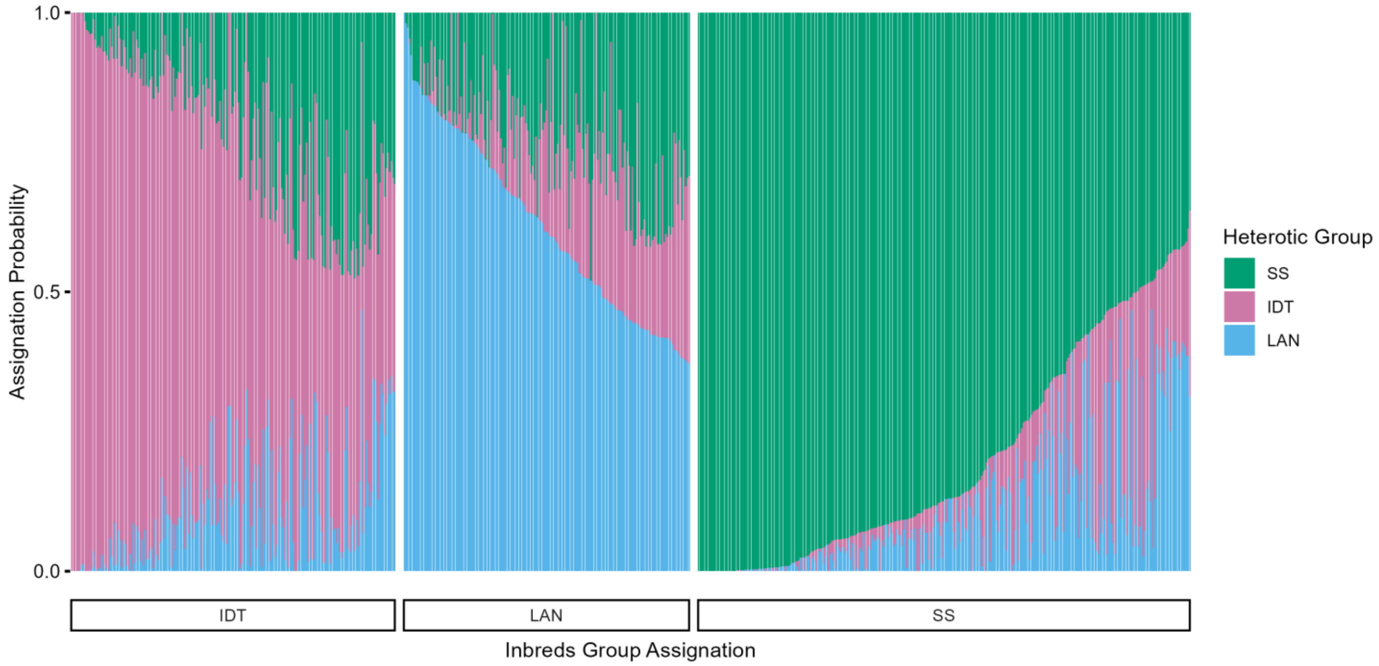

Supplementary Figure 11 *Population Structure of the dataset according to ADMIXTURE origin assignation for K = 3 and for era 2.*

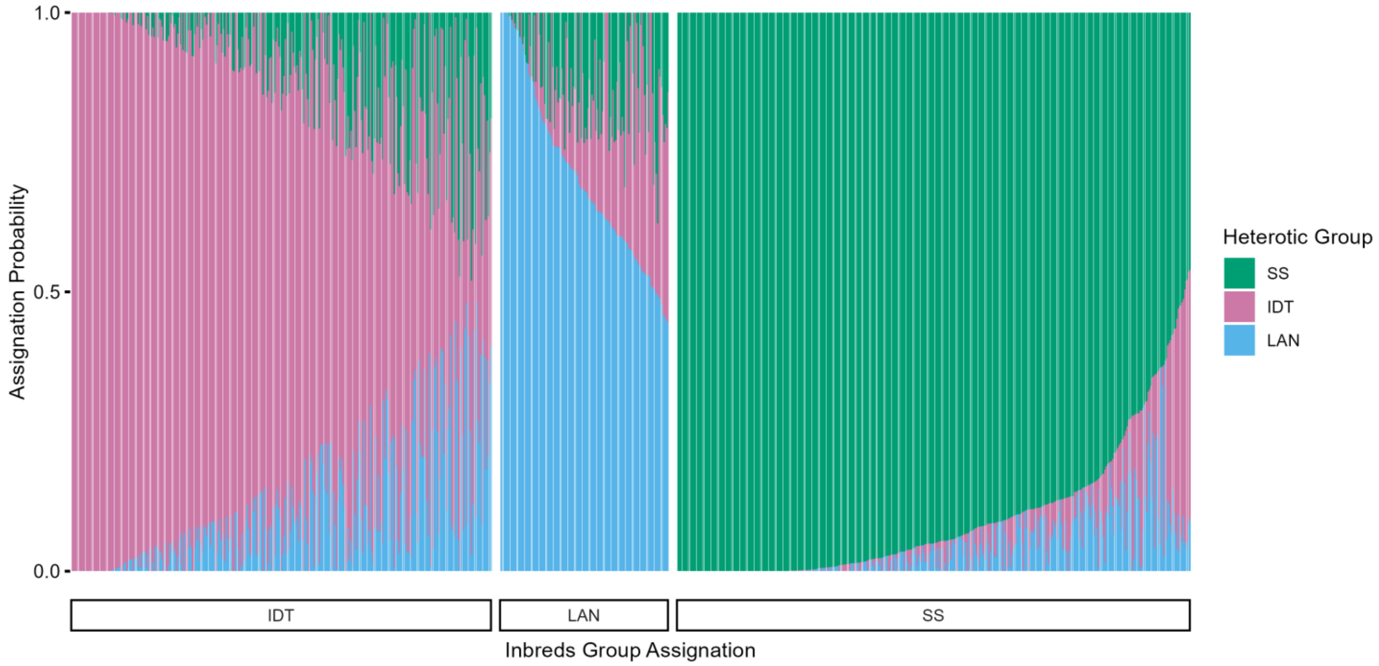

Supplementary Figure 12 *Population Structure of the dataset according to ADMIXTURE origin assignation for K = 3 and for era 3.*

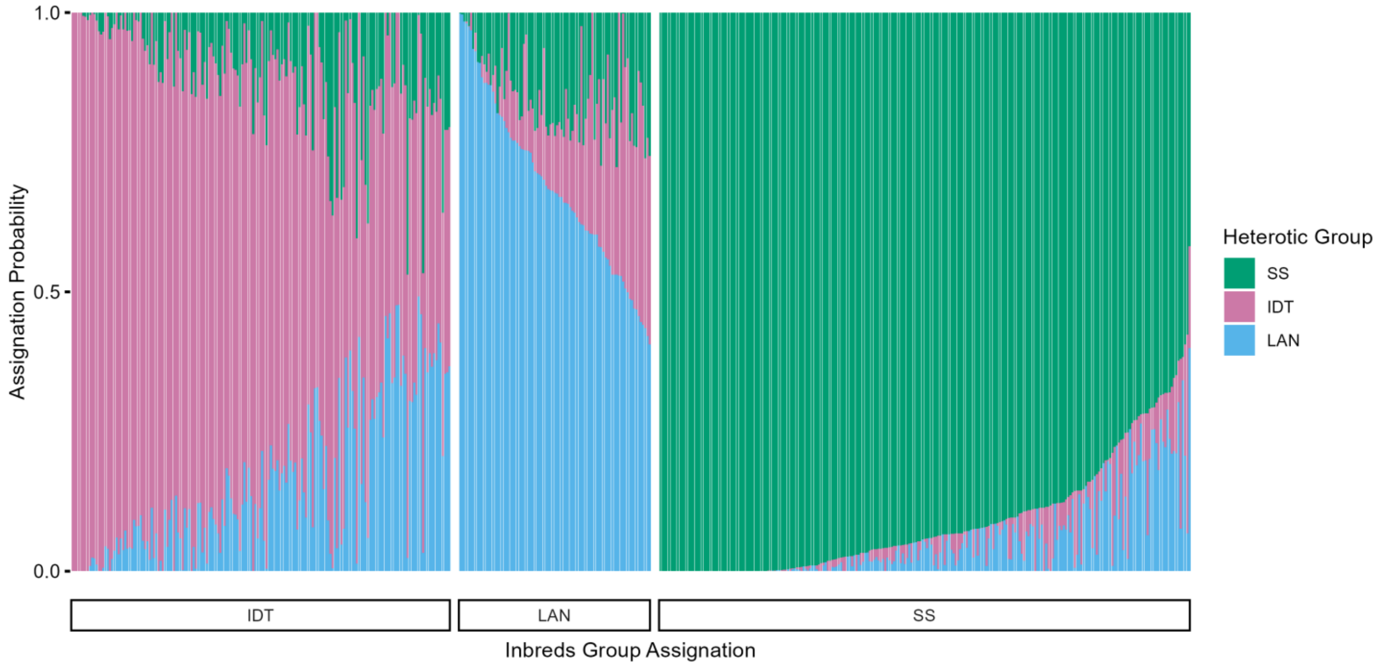

106

Supplementary Figure 13 *Population Structure of the dataset according to ADMIXTURE origin assignation for K = 3 and for era 4.*

107
